# Wearable Sensor-Based Human Activity Recognition in the Smart Healthcare System

**DOI:** 10.1155/2022/1391906

**Published:** 2022-02-24

**Authors:** Fatemeh Serpush, Mohammad Bagher Menhaj, Behrooz Masoumi, Babak Karasfi

**Affiliations:** ^1^Faculty of Computer and Information Technology Engineering, Qazvin Branch, Islamic Azad University, Qazvin, Iran; ^2^Department of Electrical Engineering, Amirkabir University of Technology, Tehran, Iran

## Abstract

Human activity recognition (HAR) has been of interest in recent years due to the growing demands in many areas. Applications of HAR include healthcare systems to monitor activities of daily living (ADL) (primarily due to the rapidly growing population of the elderly), security environments for automatic recognition of abnormal activities to notify the relevant authorities, and improve human interaction with the computer. HAR research can be classified according to the data acquisition tools (sensors or cameras), methods (handcrafted methods or deep learning methods), and the complexity of the activity. In the healthcare system, HAR based on wearable sensors is a new technology that consists of three essential parts worth examining: the location of the wearable sensor, data preprocessing (feature calculation, extraction, and selection), and the recognition methods. This survey aims to examine all aspects of HAR based on wearable sensors, thus analyzing the applications, challenges, datasets, approaches, and components. It also provides coherent categorizations, purposeful comparisons, and systematic architecture. Then, this paper performs qualitative evaluations by criteria considered in this system on the approaches and makes available comprehensive reviews of the HAR system. Therefore, this survey is more extensive and coherent than recent surveys in this field.

## 1. Introduction

Most communities are committed to preparing their healthcare systems that adapt to demographic change (as the world's aging population grows). The development of new systems with medical and assistive technologies to provide long-term care or create appropriate environments (providing living conditions with the help of the environment) shows that researchers are examining the quality of life of the elderly and their independence [1–3]. HAR is a new technology that can recognize human activity through sensors and computer systems [4–8]. HAR systems are sophisticated and can monitor individuals' situations and provide valuable tools for emergencies [9, 10]. Activities refer to behaviors [11] that consist of a sequence of actions performed by one individual or more interacting with each other. Providing accurate and appropriate information about activities is one of the most critical computational tasks in the activity recognition system [12, 13]. With the growing maturity of computing, machine learning algorithms, and neural networks, HAR based on wearable sensors [14] has become popular in various fields, including smart homes [15], healthcare for the elderly [16, 17], medical services, improving human interaction with computers, security systems, mechanization in industry, monitoring athlete training, rehabilitation systems, and robot monitoring system. It is classified into three categories in data acquisition: the external sensor (non-wearable), the wearable sensor, and a combination of the two mentioned above. In systems based on external sensors such as cameras [18], devices are installed at fixed locations where activity recognition is limited in the camera viewing angle. Two similar activities may not be detectable when we use the camera, and privacy is also violated. In recent years, wearable sensors have been considered in the healthcare system due to cost reduction, ease of use, and continuous monitoring. Wearable technology seems to be a practical step towards achieving the goal of monitoring patients at home. These systems are sophisticated and able to monitor individuals' situations and realize the object of remote monitoring of the elderly. In the HAR system (HARS), the signals obtained through wearable sensors are approximately more desirable than the signals obtained by video cameras, for the following reasons.

- Wearable sensors overcome the environmental and stationary constraints that cameras often suffer from (limitation of vision in cameras due to their fixed position).
- Placing multiple sensors in the body leads to more accurate and efficient use of the signal in the human body.
- In wearable sensors, the signals received are for a specific purpose, while the signals received by the camera may contain information from other non-target people in the scene.
- Wearable sensors observe privacy more than cameras. Video recorders continuously record the entire body during daily life activities.
- Supervisors should stay in the environment specified by the location and capabilities of the cameras throughout the day.
- The complexity and cost of video processing are other challenges in using video.

However, some wearable sensor challenges include personal satisfaction, appearance, size and comfort rate, development and support, online data acquisition and processing, energy consumption, and privacy issues. In this paper, we extract, categorize, and describe the critical challenges of the human activity detection system based on past research. We then discuss general solutions to these challenges. In addition to the challenges of data collection tools, the HARS based on wearable sensors has challenges such as knowledge extraction (variability within the classes and the similarity between types of activities), data collection (e.g., generalization, adaptability, missing information, sensor relevance, and multisensory), nature of the human activity (e.g., flexibility, the complexity of the activity, dynamics, and multiresident), and security. The most critical problems of HARS are scalability, complex actions, and human behaviors in a complex environment. These challenges illustrate the role of developing a systematic feature display method to describe the nature of activity-related signals [15, 16]. Researchers have proposed approaches to address these challenges, including handcrafted and deep learning in all HAR components. According to the classification we made in the HARS analysis, the software part is divided into preprocessing and categorizing and recognizing. The preprocessing component includes feature computation and windowing, feature extraction, and feature selection. Categorizing and recognizing can be based on supervised, unsupervised, and semisupervised learning. There are methods described in each section (ideas, advantages, and disadvantages are stated and can be seen under tables). Windowing can generally be based on time, sensor, or activity. DL methods can be used in all components of HAR, even in combination. Well-known handcrafted methods in feature extraction are linear discriminant analysis (LDA) and principal component analysis (PCA). Handcrafted methods in feature selection are divided into three categories: filtering, wrapping, and embedding. In general, the proposed methods of categorization and recognition include K-nearest neighbor (KNN), support vector machine (SVM), quadratic discriminant analysis (QDA), decision tree (DT), K-means, and hidden Markov model (HMM) which are handcrafted essential HAR methods. Deep learning (DL) methods considered included convolutional neural network (CNN), deep belief network (DBN), and recurrent neural network (RNN) (specifically long short-term memory (LSTM)). In DL, a multilayered architecture (deep) is constructed for various objects, including feature selection and classification. Each layer in the deep network performs a non-linear conversion at the previous layer's output.

This paper provided a coherent architecture for HARS and analyzed all components. Challenges of HARS have been categorized, then investigated, and discussed. We have also examined the HARS from hardware and software aspects, including components. The types of sensors and locations are checked, and public datasets obtained from wearable sensors are described and organized in the table. The types of strategies proposed by researchers have been analyzed and evaluated qualitatively. According to the comparisons that have been made with other surveyed papers, this paper has tried to examine all aspects compared to the others. We have considered a section to review the essential survey papers, and it can be seen that this paper has performed a practical and comprehensive analysis. We provided Table 1 for abbreviations and symbols contained in the text of the paper.

Section 2 examines the related work and compares the previous survey papers and this paper.  Section 3 examines some activity recognition applications.  Section 4 discusses the healthcare system's architecture and the activity recognition position.  Section 5 presents HAR challenges based on wearable sensors from different dimensions and then discusses them. In Section 6, HARS is examined and analyzed from various aspects. Finally, in Section 8, conclusions and future work are mentioned.

## 2. Relevant Surveys

This section reviews seven well-known recent survey papers between 2016 and 2021.  Table 2 shows aspects of HARS that each paper has dealt with and studied. According to Table 2, we compared recent survey papers based on architecture, challenges, datasets, sensors system, sensors types, applications, approaches, evaluations, HARS component classification, analysis of every component with table, and discussion. The value of “Yes” in some cells of Table 2 indicates that the survey paper listed in the table row has examined the criteria mentioned in the title of the column of the table, and the value of “No” means that this paper has not addressed this issue. The value of “some components” means that this paper addresses this issue somewhat. The number of figures and tables used in each survey is also mentioned.

Kumari et al. [10] examined and analyzed wearable sensor systems and types of wearable sensors. The advantages and disadvantages of wearable sensors and the components of a wireless wearable system are described. This paper details each wearable system examined, including the sensor's location on the body, the learning approach used, and the number of test settings. In [3], each step of the wearable sensor-based HAR is detailed and then feature learning, feature selection, and classification are examined. HAR based on camera-based systems and systems that combine wearable and ambient sensors are also reviewed in this paper. It also outlines the applications and challenges of HAR. In [12, 13], sensor-based HAR and deep learning are important. This paper examines the HARS from three aspects: sensor method, deep model, and application, and describes the purposeful challenges that will lead to future research. In [19], the types of activities are defined and the differences in the types of activities are discussed. In [20], HAR methods have been classified, and some of the advantages and disadvantages of the methods are given. HAR methods are classified into two main groups based on how the data are generated: sensor-based HAR and vision-based HAR. Then, it describes each group and the HAR process. It also discusses the challenges and methods of deep learning. Ramanujam et al. [21] discussed deep learning techniques and wearable sensor-based HARS. Also, the advantages and disadvantages of some methods are described, and various conventional datasets are discussed. In [22], research work on HARS using different sensor technologies has been reviewed. This paper identifies the limitations concerning the hardware and software characteristics of each sensor type. It compares them with the benchmark characteristics retrieved from the research work presented in this survey. Finally, it concludes with general statements and provides future research guidelines for HAR in the sensor classification.

According to the above sentences, a review paper that covers all the components related to the HARS seems essential. We examined all aspects and components related to the HARS, as shown in Table 2.

## 3. Application of HAR

**Figure 1:**
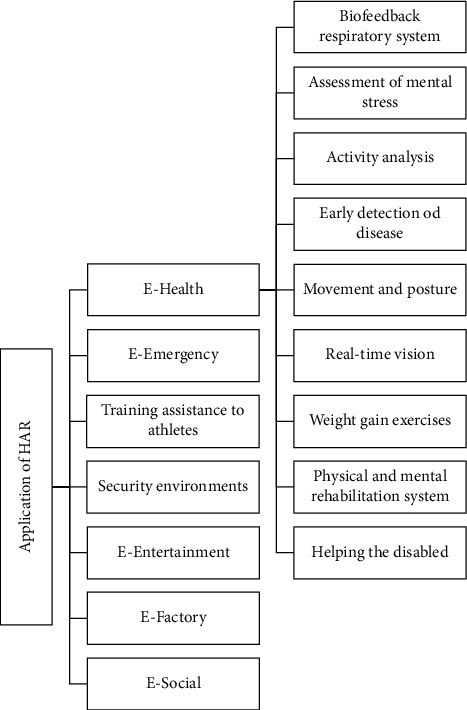
Categorization for HAR applications.

Figure 1 provides a classification of HAR applications briefly described below.

- **E-health** includes remote care and control of the person (such as the elderly), physical and mental rehabilitation, activity examination, respiratory biofeedback system, mental stress assessment, rehabilitation system, weight training exercises, real-time vision, movement, and posture. An e-healthcare system can enable individuals with disabilities to live longer independently. Using “a simple button for sudden anxiety and fear,“ “personal alarm devices for the elderly,“ and “cell phones with a panic button“ can provide psychological peace for the elderly and security for family members and friends [10].
- **E-emergency** includes monitoring people in dangerous places like earthquakes, landslides, and fires.
- Training assistance to athletes focuses on e-fitness programs, health, organizational systems for fitness clubs, and athlete health.
- Security environments include monitoring, intrusion detection, and automatically recognizing abnormal activities.
- **E-entertainment** is mainly related to human and computer interaction aimed at face recognition, situation, gesture, and real-time HAR.
- **E-factory** includes monitoring operations, worker protections, and cooperation between workers.
- **E-sociality** includes recognizing emotions and pressure to discover social relations between people [23].

It is estimated that the number of elderly citizens will increase significantly in the next decade. Health issues among older people are a significant concern in developed countries and developing economies such as Brazil and India. Elderly citizens occupy a large part of health-related facilities due to health issues. In the traditional healthcare system, needs are not entirely met due to the increase in population. On the other hand, medical services are not accessible and affordable. Therefore, HAR is fit for the healthcare of the elderly remotely.

## 4. Smart Healthcare System

**Figure 2:**
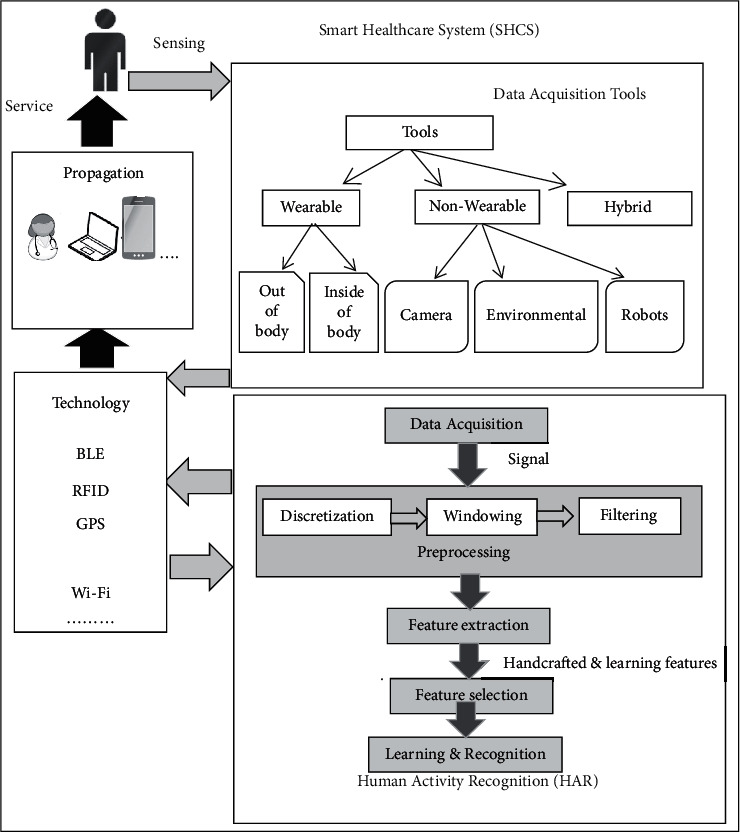
HAR in SHCS for monitoring the elderly.

Figure 2 presents the smart healthcare system (SHCS) architecture for monitoring human activity, including five independent components: data acquisition tools, HAR, technology, propagation, and service. Sensor-based activity recognition is essential in many applications, including care for the elderly and intelligent healthcare [18–21]. Activity recognition in the SHCS is a way to facilitate healthcare for treatment and care of patients, reduce the workload of health personnel, reduce costs, and improve the quality of elderly life.

Medical experts believe automatic activity recognition is one of the best ways to identify and discover new medical conditions to monitor daily activities [7].

HAR consists of five main components: data acquisition, preprocessing, feature extraction, feature selection, and learning and recognition. According to Figure 2, preprocessing is a critical step in data processing, including discrimination, windowing, and filtering. First, the signals are discretized, and time and frequency characteristics are considered for them, and these characteristics are widely used to calculate the feature. Mean, median, and variance are the characteristics of the time domain.

Then windowing techniques are used to split the sensor signals into parts [24, 25]. The most effective window-based methods are activity-based, time-based, sensor-based, latest-sensor-based, and sensor-dependent. In activity-based windowing, data are separated at the point of activity change. In a time-based window, activity data streams are divided into static windows. In the sensor-based window sequence *s*_1_, *s*_2_,…, *s*_N_ is separated into windows with the equal number of sensor events *w*_1_, *w*_2_,…, *w*_*M*_, and the *w*_1_ window is signified by [si- Δs, si]. The results of the window length vary from one window to another. In time dependence, two sensor events broadcast separately may be part of the same window [26, 27]. The filtering process helps to replace missing values and remove outliner values. The HAR component includes feature extraction and selection and then learning and recognition. Data mining is the process of modeling data to extract hidden knowledge. Feature extraction from raw data is performed using split and classification algorithms from each window, respectively. Feature extraction is done linearly and non-linearly to reduce the dimension, referred to as LDA and PCA methods. Then, value-based attributes have been selected that increase the accuracy of activity detection. Feature selection methods include filtering (canonical correlation analysis (CCA) [28]), wrapper (such as SVM and neural network (NN)), and embedded method [29].

It should be noted that the collected data must be transferred to the HAR component for analysis using technologies such as Wi-Fi. Detected activity can be propagated by technology. In case of any problem, the person will be served. It all depends on the new hardware platform. All the components mentioned in this architecture (Figure 2) will be discussed clearly in the following sections.

## 5. Challenge of HAR

There are challenges to activity recognition in the SHCS, and researchers have presented methods to address these challenges. These methods can be separated into two general categories: preprocessing and classification stages.

**Figure 3:**
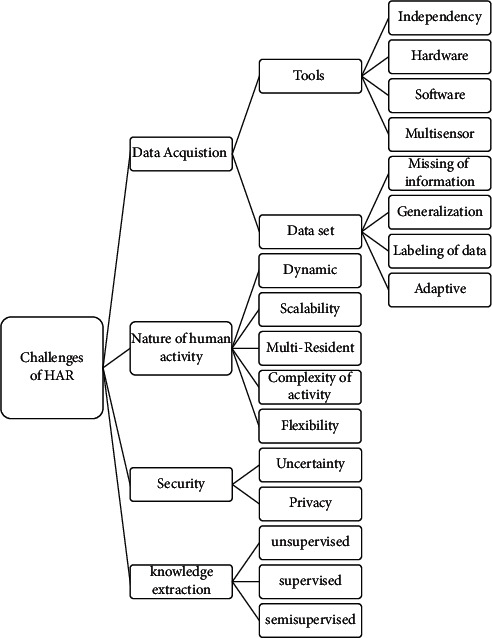
Proposed classification to recognize human activity in the SHCS.

In the preprocessing phase for windowing, the expansion of the sensor dependence on the information and the two sensors' mutual sensitivity depend on the occurrence of a pair of sensors in the total current. There is a dependency between the two sensors [27]. Different methods are presented for windowing due to their importance and influential role in HAR. Activity-based learning is not appropriate for online recognition because it delays coming data decisions; this windowing is ideal for labeling data. Many errors in time windowing-based classification are due to incorrect window length selection. Sensor-based learning can be complex when two or more people live in a smart home. It is easy to have a linear hyperplane between two classes in the SVM classifier. Deep NN methods are of great interest in pattern recognition in many applications. In particular, CNN and LSTM are the most potent DL methods used in activity recognition [30].  Figure 3 provides the proposed classification for the challenges of activity recognition in SHCS using wearable sensors described as follows.

### 5.1. Data Acquisition

Data acquisition is facing hardware and software limitations, including low-power and lightweight tools and security. Sensory data are inherently noisy and involve varying sampling rates and complex correlations that data cleaning techniques are needed to reduce these effects. These techniques filter and delete inappropriate information to preserve only the relevant information [31]. Data acquisition challenges are related to tools and datasets, which we will describe in the following sections.

#### 5.1.1. Tools

**Figure 4:**
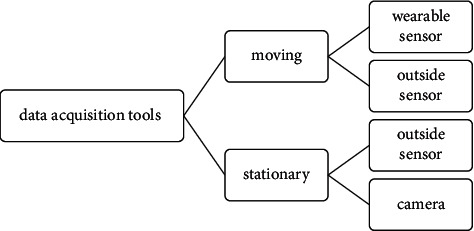
Categorization of data acquisition tools.

Data acquisition tools can generally be moving or stationary, as shown in Figure 4. The wearable sensor is movable, and the outside sensor can be moving or stationary, and the camera is stationary. In each of these tools, it is possible to receive online and offline data. In the following, we will examine the challenges of wearable sensors and cameras. Wearable sensors have vital challenges such as individual satisfaction, power consumption, and wireless system involvement described in the following.

- **Thermal damage:** the possibility of thermal damage to the human body should be considered. By controlling the frequency of the wireless sensor, the cycle of radio effects from the wearable sensor should be reduced. According to the human body and design parameters, a new framework has recently been suggested to consider health and sustainability needs.
- **Appearance and size:** many related companies make every effort to produce acceptable sensors for the individual. Hence, they use the potential of the fashion industry. The wearable sensor's length should be such that it fits easily on the human body.
- **Water-resistant:** the wearable sensor must be with the human body everywhere for continuous monitoring and must be resistant to temperature, water, humidity, and sweat.
- **Power consumption:** wearable sensors have batteries and require very high energy to monitor humans 24 hours a day. The communication system consumes more energy than the rest. A wearable sensor can be equipped with a low-power control circuit with an energy removal mechanism, assuming the sensor is wireless and automatic. The circuit can transfer maximum electrical power from solar energy to supply the wireless sensor node. Reducing these devices' power consumption requires impressive structures in the microcontroller unit and operating system algorithms [32].
- **Wireless communication:** wireless connection is essential for continuous monitoring, as the wearer cannot move quickly with wires, and the individual is not comfortable. Alternatively, it may be necessary to communicate with more than one device. Furthermore, significant challenges are real-time data delivery, packet loss, and data transmittance disappearance.
- **Operating system:** another challenge is that wearable sensors sometimes require a particular operating system, namely, a smartwatch to extend a mobile phone requires implementing a specific operating system. With the advancement of technology, wearable systems have made significant progress. For example, clothes or other conductive fabrics can be washed like regular fabrics. Therefore, one of the critical features of wearable sensors should be that they are water-resistant because the sensor must always be with the person. It can be said that computer fabrics are the next generation of wearable devices without the need for electronic connection to the body.
- **Installation and maintenance** of wearable sensors are expensive.
- Some activities, such as walking, are challenging to detect with machine vision embedded in wearable sensors. In other words, due to a lack of information, it is not easy to see movements [10, 33].

The camera is also a traditional tool to get information carefully. Sufficient two-dimensional information is provided from different viewpoints to extract three-dimensional human movements, and the environments are predetermined. The field of view of fixed cameras is limited [31]. Other disadvantages of cameras include the fact that many people do not feel satisfied that all their movements are frequently under control, and the issue of privacy is discussed. Also, in terms of complexity, information processing is costly.

#### 5.1.2. Dataset

The wearable device satisfies the long-term usability of a monitored environment [6]. After processing the data collected from wearable sensors by HARS, the information is made available to patients, caregivers, consumers, and healthcare professionals. People (patients or elderly) should be encouraged to live independently by improving remote monitoring and interaction. But datasets face challenges such as noise and loss.

### 5.2. Nature of Human Activities

The fundamental HAR system problems are scalability [34], complexity of human activities, and behaviors in a complex environment [15, 32]. Wearable sensors, including computer vision, have met this challenge to some extent and perform well in detecting complex activities. Compared to walking detection, which requires little knowledge, more research has focused on activity recognition related to interaction with other people or objects. Because many activities are naturally identified with interactions, techniques often depend on recognizing them from a whole perspective. Some methods only want to recognize an activity such as walking, but some work more powerfully and deal with human interactions with an object. Humans manipulate an object over time or damage it altogether, making it difficult to detect. Even if the object is open or closed like a refrigerator, it is entirely different. A person's specific activity may change over time which is a challenge [35]. There are many technical challenges in designing activity monitoring systems. A person can perform a specific activity in different ways in various situations; also, different activities may be done in the same way. Uncertainty significantly reduces the accuracy of the recognition [33]. The healthcare system uses wearable wireless sensors to overcome some of these challenges, where continuous patient monitoring is possible without even hospitalization. There are several programs to monitor activities in real time. In clinical programs, continuous monitoring of individuals' physical and mental states is essential for their safety [10].

### 5.3. Security

Despite significant efforts in the HARS, it still suffers scalability, security, and privacy. In the system, a large amount of collected data can be used in various fields, but at the same time, it can cause several security issues. In SHCS, security and privacy are currently very complex issues, and also, the increase in the number of sensors and devices led to more challenges in this area [10]. In HARS, a large amount of data is received from sensors at any time. There is a risk of eavesdropping and hijacking attacks in communication channels, so there is a possibility of violation of privacy and data security. Recently, blockchain technology has been used to provide a reliable and efficient system in the Industrial Internet of Things (IIoT) (such SHCS) [36].

### 5.4. Knowledge Extraction

Today, learning and understanding human activities have a special place in many research fields. HAR comes with many challenges depending on the type of activity, environment, and person [37]. Identifying these challenges will lead to some barriers to the activity recognition system. Since potential models used in HAR require labeled datasets, different system configurations are problematic. An active learning method based on irregular sampling has been proposed to deal with this issue in a low-cost manner. With active learning, annotations may be reduced by selecting only the critical information points. Labeled samples are needed for training to set parameters in possible models of HAR. Smart homes with different designs create two limitations:

- House designs modifications and differences in performing activities by different people are an issue. The trained model based on set parameters of one particular home cannot be used for another home with a different design.
- Given that it is possible for a person to change activity over time, the trained model will lose its effectiveness. Although this problem can be partially solved with additional information, its implementation is costly and not operational. Instead, DL methods and NNs address these problems. Therefore, installing large-scale activity recognition systems with diverse designs and residents is possible. This scalability provides a solution to deal with the consequences for the elderly population [34].

Knowledge extraction and activity recognition challenges are generally divided into three types: supervised learning, semisupervised learning, and unsupervised learning, which we will examine separately in the following sections.

#### 5.4.1. Supervised Learning

Numerous studies have focused on wearable sensor-based HAR using supervised learning to achieve the desired results but require labeled datasets. Labeling each instance in supervised learning methods is expensive and requires a lot of human effort. Some datasets provided by a human may ignore user annotations. This labeling should be updated each time a new activity is added, so in these cases, semisupervised or unsupervised learning methods are more widespread [38, 39]. Tracking a predefined list of activities requires a significant amount of training data. Collecting, labeling, and annotating data in an intelligent environment is time-consuming and error-prone. There are always challenges between the accuracy of the annotation and the time required for annotation. It is a need to find methods to reduce the time of data labeling and provide acceptable accuracy. It seems that using semisupervised or unsupervised approaches instead of supervised techniques to identify normal daily activities in the smart home environment is appropriate [9]. The use of unsupervised algorithms allows them to overcome issues related to labeled data, thus making big data analysis easier [40]. Another problem with HARS is the process of learning new activities. Sometimes inexperienced or very old people are used to collecting data in laboratories, so the training and learning process is inaccurate and sometimes even fails. This challenge reduces accuracy and compatibility in specialized systems.

On the other hand, the lack of training makes known activities the same for each user and eliminates the system customization options [16]. Class distribution is another challenge. Thus, the small number of samples and the imbalance in residents' activities in smart homes lead to step-by-step decreased efficiency and accuracy of learning methods. The class overlap is another challenge of sensory information in an intelligent environment. This problem may lead to ambiguity [41]. Most low-performance machine learning algorithms are characterized by such issues [7]. There are several ways to deal with this problem: sampling, sample reweight, cost-sensitive learning, or creating a specialized algorithm. The SVM has shown its ability in an unbalanced dataset because it only considers the support vector. This classifier is computationally efficient and can achieve good performance at high differences between classes and low differences [42].

#### 5.4.2. Unsupervised Learning

Unsupervised learning methods are less accurate than supervised. The number of clusters and the number of activities may differ; thus, a most similar cluster to the relevant activity should be selected from the existing clusters [23, 43]. The unsupervised learning algorithm can recognize activities without labeled data and use the generated data in actual cases. Various sensors can improve gesture recognition, including a ring on the finger and a bracelet on the wrist [40].

#### 5.4.3. Semisupervised Learning

Some research studies analyzed the performance of semisupervised learning methods in healthcare applications that train only a small amount of data and many unlabeled samples, thus reducing cost. Classic semisupervised training independently uses two types of classification and allows data to be updated using highly reliable unlabeled samples. In unsupervised methods, samples selected initially for clusters often lead to dangerous consequences such as low accuracy. On the other hand, semisupervised and unsupervised approaches in real life are more desirable with more uncertainty. Therefore, solving complexity and accuracy is challenging and often leads to erroneous predictions [38].

### 5.5. Discussion

In this section, we review techniques to address some of the challenges of HARS in the SHCS. Due to the importance of windowing and its influential role in accuracy, various methods for windowing have been proposed, but according to studies, this part still faces challenges [44]. Time-based windowing is considered due to the simplicity of the HARS, but due to the incorrect choice of window length, it causes errors and reduces the accuracy of recognition. Recursive neural networks (including LSTM) play an influential role in mitigating this challenge. Sensor-based learner encounters problems when two or more people live in a smart home due to information interdependence. When multiple wearable sensors are used, the rate of occurrence of the sensors in the total current and the two sensors' mutual sensitivity can be considered a problem. There are methods for extracting knowledge and recognition (supervised, unsupervised, and semisupervised), each facing challenges.

Some methods are used in combination to meet some of these challenges. Feature extraction and selection also play a unique role in increasing the performance of the HARS. Features can be extracted by handcrafting and deep learning techniques (such as filters in the CNN method) that will significantly impact when used in combination. In the SVM method, low-dimensional input space can be converted to a higher-dimensional space with functions that lead to better class separation. Deep NN methods have made significant progress in pattern recognition in many areas. CNN, in particular, is one of the most potent deep methods widely used in recognizing activity based on time series [45]. Researchers generally pay special attention to DL and fuzzy computing to address some of the challenges associated with windowing, feature selection, and recognition. Activity-based learning is not suitable for online recognition because it has to wait for decisions about future data. This method is more suitable for labeling data. Activities are classified into two classes, simple and complex. Complex activity is a sequence of simple activities, so there is a problem in recognition. Long short-term memory networks play a significant role in this. These networks can better play a role in feature selection and activity recognition by incorporating previous results into current decisions. Flexibility is another characteristic of human activities that is considered a challenge in recognition over time. A fuzzy inference system and ontology can be effective in meeting this challenge.

## 6. HAR Analysis

**Figure 5:**
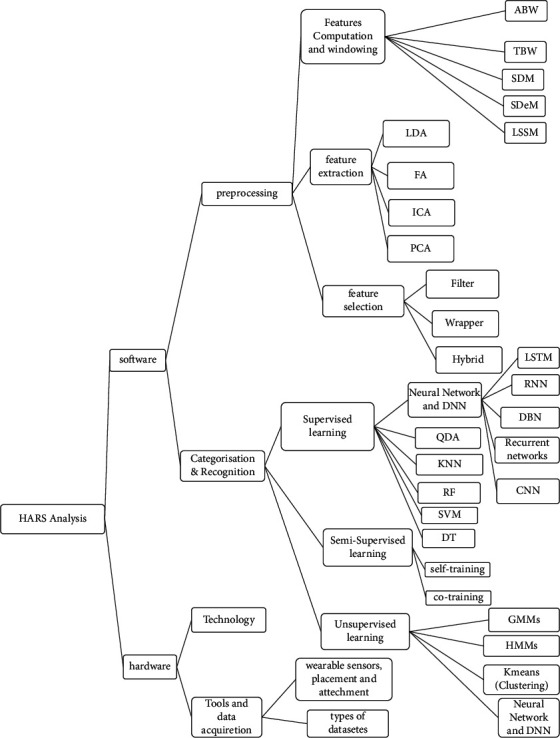
HARS' main component categorization.

HAR based on wearable devices (including sensors and accessories) is one of the most critical issues in the present age (smartphones are an example). Recognizing and monitoring human activities is essential for providing healthcare services and assistance to the elderly and people with physical or mental disabilities. Due to their disability, they should be monitored to avoid being in abnormal situations (like a fall) and their consequences.  Figure 5 provides a classification described and analyzed in the following sections.

### 6.1. Hardware

#### 6.1.1. Tools and Data Acquisition

In this section, wearable sensors and their components will be reviewed first, and then places of sensors and how to attach them to the body are checked.

**Figure 6:**
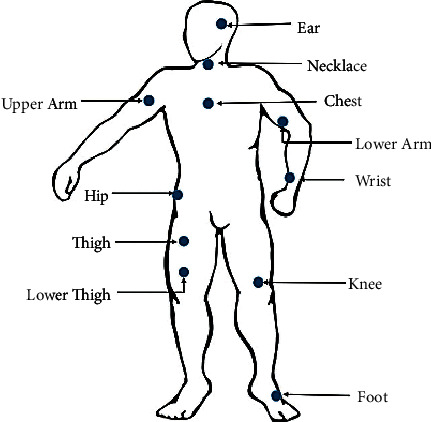
Different common locations for wearable sensors [22, 23, 43].

*Wearable Sensors, Placement, and Attachment*. Wearable sensors are the newest HAR tools in the present age, placed in various human body parts. It is important to know the suitable location of the body for wearable sensors and the right tools to attach them to the human body. The sensor location on the body has a significant effect on measuring body movements and recognizing activity, so much research is being done in this area. According to Figure 6, examples of the body parts where the sensors can be placed are visible and are usually located on the sternum, waist, and belt. Wearing sensors around the waist placement can monitor human movements more accurately because it is close to the human body center. The number of sensors, such as the sensor's location in the HARS, is essential. According to research, the combination of the chest, ankles, and thighs to embed the sensor is the most accurate. The results show that applying the accelerometer to the upper torso and lower torso simultaneously improves HAR accuracy [22, 23].

The accelerometer is the best sensor for activity recognition. But when the accelerometers along with gyroscopes, magnetometers, and sphygmomanometers are used in the system, the performance is improved. Smartphones often incorporate all types of sensors [16].

**Figure 7:**
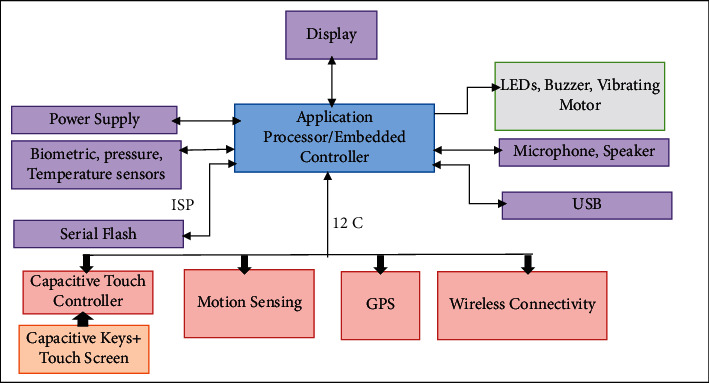
Diagram of a wireless wearable sensor block [10].

Wearable technology seems to be a practical step towards checking the elderly and patients at home. These systems are sophisticated, monitor individual situations, and provide valuable tools for emergencies [10]. Wearable sensors are usually small and wireless enclosed in a bandage or some patch or something covered. These objects could be a ring, a shirt, patches of skin, a watch, nails, or hair. Due to physical activity, cataloging of human life activities using wearable sensors has been extensively studied. The wearable system architecture can be seen in Figure 7, consisting of a power supply, screen, wireless connection, motion sensor, and software processor blocks. Accelerometer, magnetometer, and gyroscope are among the most commonly used wearable sensors (motion sensors or microelectromechanical systems (MEMS)). In addition to motion sensors, biometric sensors can detect vital signs, referred to as EEG and EMG. Analogue front end (AFE) tasks include preprocessing sensor information with filters and converters. Devices such as mobile phones can use a combination of sensors such as gyroscopes, accelerometers, microphones, and even cameras and other technologies. Accelerometer applications include step counting and person-oriented changes used in smartphones today. Despite progress in this area, there are still limitations, such as hardware. For example, battery limit and resource consumption should be considered [43–45]. Sensors are considered the most popular interface between the user and the device, including elements for alerting the user. Pulse width modulation (PWM) leads to the stimulation of these elements. In addition to the hardware, the wearable system software depends on the device features, including the processor [10]. Different sensors are used to monitor HAR in smart homes, and there are different perspectives on their classification. Sensors can be classified as discrete sensors such as passive infrared (PI) sensors, contact switch sensors (CSSs), and radio frequency identification (RFID) with binary output.

**Figure 8:**
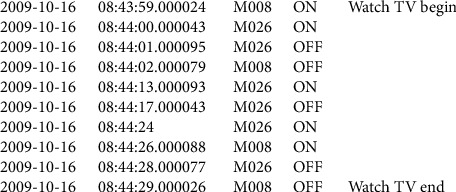
Sample of raw data of the wearable sensor.

**Figure 9:**
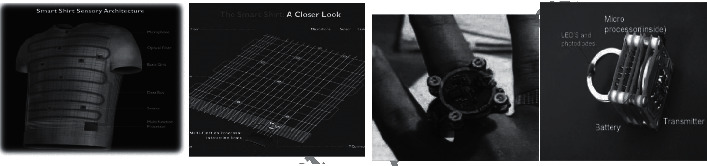
Overview of a smart shirt and ring sensor [10].

In contrast to discrete sensors, continuous sensors include physiological, environmental, and multimedia sensors with simple or complex information flow such as natural numbers, images, or sound. In a manner, the sensors are wearable or peripheral. Inertia (accelerometers and gyroscopes) and vital sign sensors (biosensors) are wearable sensors. People use wearable sensors to generate more information about their position, movement, location, and interaction. Peripheral sensors achieve information about the smart home environment, such as temperature, humidity, light, pressure, sound, and so on. They are not made to monitor group activities and discriminate between residents' movements or actions [33]. Several sensors can perform various monitoring tasks to measure properties such as movement, position, temperature, and ECG. An example of the binary collected data is shown in Figure 8. The smart shirt and ring sensor shown in Figure 9 are examples of wearable sensors. A smart shirt is a device that uses optical and electrical fibers to check some essential organs of the human, such as respiration speed, body temperature, inhalation measurement, and so on. The ring sensor is a pulse oximetry sensor based on a biosensor sensor and monitors heart rate [46] and oxygen saturation. Among the wearable sensors, we can mention biosensors, which include natural sensory elements and transducers, and the collected data from these wearable sensors are processed according to a particular program.

These sensors have overcome some of the limitations of traditional tools and provided the ability to control people remotely (for people with serious illnesses such as Parkinson's disease or heart attack) [10].

In contrast to wearable sensors, cameras also act as external receiving devices for HAR. Distinguishing activities and movements from video sequences has been a significant focus of research. According to the study, activities such as sitting, moving back and forward, and rotating by video within the range defined for the camera are well recognized. But two similar activities may not be distinguishable. Therefore, it is necessary to use facts about personal behaviors to build a movement model. It can be said that the combination of camera and wearable sensors has a significant result of the HAR accuracy and status detection. In HAR, the signals obtained through wearable sensors are approximately more desirable than those received by video [31].

*Types of Datasets.* There are various datasets for HAR based on wearable sensors, and in this section, we will review the popular datasets that most researchers used to evaluate the proposed methods.  Table 3 describes these datasets with their details, and we will review the mentioned datasets in the following.

*Opportunity*. It is general and consequential, especially for complex activities with multiple wearable sensors [13, 47]. A number of participants (PN) performed various activities for 6 hours with sensors such as gyroscope, accelerometers, and magnetic sensors (IMU) to obtain this dataset. The number of these activities is 17. These sensors collect information in three dimensions (3D) and various numbers. Participants had 5 ADL sessions and one practice session. Data are considered in multilayer, high-level activities, medium-level activities (such as arm movements), low-level activities (right and left-hand movements and use of objects), and actions [47].

*DLAs*. This dataset is received from three sensors, each with a three-axis accelerometer and a three-axis gyroscope. On the other hand, the proposed system intends to identify 13 activities. The dataset was received from 23 volunteers with wearable sensors, consisting of 13 men and ten women aged 27 to 34. These activities include walking, sitting, standing, and any routine human activity [9].

*UCI*. This dataset [48] focuses on repetitive daily activities, including static activities, dynamic activities, and switching between activities that often follow each other. The tool used to collect data from the human body is the Samsung smartphone, which includes three-axis sensors and records information at 50 Hz.

*PAMAP2*. This dataset includes long and repetitive physical activities commonly used for systems to describe energy consumption [49]. It consists of the most complex activities such as cycling and football, with 18 activities recorded by nine people. Several sensor samples have been used to collect data suitable for activity detection algorithms [50, 51].

*SBHAR.* Smartphone-based HAR (SBHAR) dataset is based on a group of 30 people using a smartphone that includes a gyroscope and two accelerometers. This dataset supports six activities and contains the transfer information needed to evaluate the system [51, 52].

*Mhealth.* Mobile health (Mhealth) includes body movements and vital signs recorded with several activities. The sensors such as acceleration and magnetic are positioned on the chest, right wrist, and left ankle of ten volunteers and are used to measure movement in different parts of the body. The chest-mounted sensor also offers 2-lead ECG measurements, which could monitor the heart and examine various types of arthritis or the effects of sport on the ECG. This dataset contains a fine-grained real-time sensor that studies activities at small time intervals with no specific time symbols or locations in the dataset [53, 54]. The volunteers' movements and vital signs during several physical activities and rest time between them were measured using wearable sensors [55].

*WISDM.* This dataset includes almost simple activities such as walking, with sensors built into a smartphone. Participants put a smartphone in their pocket to record activities. During these activities, the sampling rate of the 20 Hz accelerometer sensor was maintained [56].

*REALDISP*. REAListic sensor DISPlacement (REALDISP) was initially accumulated to study sensor movement effect in the real-world HAR process. This dataset is based on ideal placement, self-placement, and induced displacement. Ideal and reciprocal displacement conditions represent the types of intense shifts and describe boundary conditions for recognition algorithms. In contrast, self-placement reflects users' perceptions of how sensors are connected, for example, in a sports program or lifestyle. The dataset includes 33 fitness activities (warm-up, cooling-down, and fitness exercises), sensor techniques (acceleration, rotation speed, magnetic field, and quaternions), and participants (seven females and ten males). In addition to examining sensor displacement, the dataset is also used to test activity detection techniques under ideal conditions [57].

*MobiAct*. This publicly available dataset includes participants' mobile data in various activities and a range of falls. It has already been published under the title MobiFall. Since MobiFall consists of multiple activities from everyday life, it also makes it suitable for recognizing human actions. MobiAct includes four different fall types and nine other ADLs from 57 participants, with over 2500 tests recorded with a smartphone. Daily life activities are selected based on the following criteria. (a) Activities that are initially falling and finally motionless, such as sitting in a chair or car step in and car step out. (B) Sudden or rapid, fall-like activities such as jumping and jogging. (C) The most common daily activities such as walking, standing, climbing, and descending stairs (“climbing stairs“ and “coming down the stairs“). The dataset's aims are complex daily activity recognition and, ultimately, behavior and fall detection. As a result, MobiAct is appropriate for crash detection and HAR [58].

#### 6.1.2. Technology

**Figure 10:**
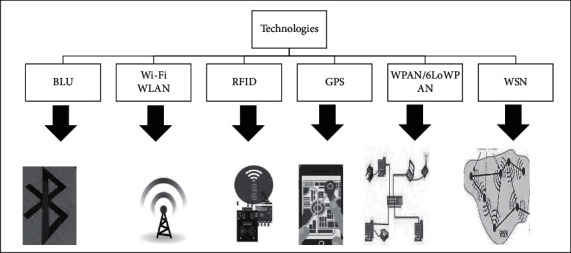
Technologies for sending data.

After the data acquisition tool collects its information, it must be sent to the HAR system using Bluetooth, Wi-Fi [20], etc. Then, after recognizing the activity, using these technologies, the information related to the recognized activity is sent to the publishing component (the supervisor can be a person, a computer system, or even a mobile phone).  Figure 10 shows the technologies that are widely used.

### 6.2. Software

#### 6.2.1. Preprocessing

Filtering data, detecting missing or out-of-range values and modifying them, and then extracting the features are done in the data preprocessing step, which is one of the main components of HAR. Segmenting the signals received sequentially to extract features from the raw data is necessary. The preprocessing operation is then applied to each section. Several ways to perform the windowing operation include creating time-based windowing (fixed-length) or changing activity at a specific point. The windowing approach with time-based windowing is suitable for online mode because it does not require additional information and preprocessing [27]. A HARS can act online or offline. In an online manner, sensory data changes in each diagnosis must be analyzed, and it must be decided about changes in the activity type [59, 60]. In this case, it can use previous sensor data to make decisions without waiting for the future. An online HARS is required by reading data from a sensor to provide an automated monitoring system for different human needs, many techniques of which are not appropriate for building an online system. In the following, we will examine the preprocessing steps.

*Feature Computation and Windowing*. Features are inputs to machine learning classifiers extracted from raw sensor data in three ways: the first method uses handcrafted features that are based on domain knowledge; the second method uses automatic extraction of learning features by deep networks [29]; and the third method is a combination of the two methods mentioned. Standard features that are part of the time and frequency domain can be extracted from the signals. Among the characteristics of the time domain when used a lot are the mean and variance.

Features such as spectral entropy and discrete Fourier transform (DFT) are part of the frequency domain used widely. One of the advantages of handcrafted methods for calculating features is that they are less expensive in computing and implementation [22, 58, 61, 62]. However, these features are widely used but are not exploratory and activity-dependent [12, 41].

**Figure 11:**
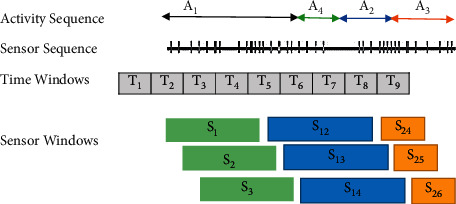
TSW and SEW methods in the preprocessing stage [33].

Various methods can be used to split sensor data to identify activities such as point change detection (CPD), time slice-based windowing (TSW), and sensor event-based windowing (SEW). CPD is an unsupervised segmentation, and the idea is to achieve sudden changes in time series and detect similar activity boundaries in real time. TSW is widely used in the cognition of physical activity. SEW consists of the same number of event sensors and parts of the data stream in sequence.  Figure 11 shows an overview of the TSW and SEW.

In some cases (for example, using supervised learning), data annotation is done at this stage. Accurate annotation of activities is essential to estimate the performance of diagnostic models. Annotation methods are separated into offline and online methods [33].

Classified datasets are divided into smaller windows (the signals from the sensors are divided into shorter sequences called windows), which is more challenging because the long windows have been shown to produce better results [52].  Table 4 analyzes the proposed windowing methods, and each case is briefly reviewed below.

*Activity-Based Windowing.* In activity-based windowing (ABW), the data stream of events is divided into windows at activity change detection points. If the length of the window is considered variable for different activities, activity recognition would be better because various activities are different in terms of complexity and execution time. Given that the boundaries of activities are not well defined due to a lack of proper definition, this will negatively impact windowing. On the other hand, finding breakpoints in the training phase requires complex calculations and low efficiency for online identification. Therefore, this method is more suitable for labeling samples because it will need consecutive data to select the next window [27].

*Time-Based Windowing.* In time-based windowing (TBW), event data streams are separated into windows with fixed time intervals. This method is used to segment signals due to its simplicity of implementation [26]. Nevertheless, multiple recognition errors in this method are due to incorrect window length selection. Window with small length leads likely contains insufficient information to make a decision. Conversely, in windowing with considerable size, multiple activity information is in one window. As a result, the time window shows more than an activity, which strongly influences decision making [27].

**Figure 12:**
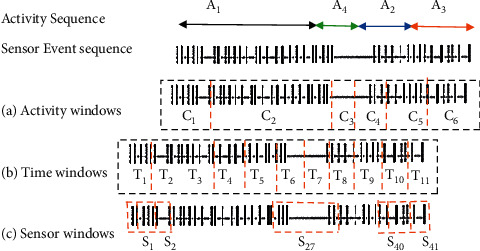
Data stream segmentation (methods) [27].

*Sensor-Dependent Windowing*. In sensor-dependent windowing (SDW), data are divided into windows of the same number of sensor events. In Figure 12(c), the sensor windows are obtained using a sliding window of sensor events of length 6. The results of the duration of the windows vary from one window to another. Multiple sensors may fire during activities, while many do not fire during off periods. According to section S27 in Figure 12(c), it can be seen that this method faces challenges. Delays between events lead to challenges such as lack of communication.

On the other hand, there may be more than one resident in the smart home, meaning that one section's information may be related to an event from one resident and the other from two residents [17]. Although this method addresses these challenges, modifications are always needed to establish the connection between sensor events. This method has advantages over the ABW, such as computational advantages, but a window containing a sensor event may take a long time. Also, processing the entire sensor event in a large window does not seem correct and takes long. Weighting is necessary because the effect of activity in the past and the start of activity now are not the same. Another challenge is when the event window includes the transition between two activities, which is necessary to describe the previous event. There may be no connection between the two activities, which can also be weighed to solve this problem [27].

*Sensor Dependency Extension Windowing*. In the sensor dependency extension windowing (SDEW), the two sensors' related data depend on the order in which a pair of sensors occurs in the whole data stream. Multiple sensors can be installed to recognize a specific activity. Sequences in sensors can be s1s2s3s4 or, secondly, s1s3s2s4 to do an activity [17]. These two sequences also lead to a similar activity, and it can be a dependence between sensors S1 and S2. A small amount of dependency between these sensors will be lost in several cases. Also, there are regularly executed activities in equivalence, and the sensor events of one activity can be described for other information. Out-of-date cross-information cannot consider this condition [25].

*Last-State Sensor Windowing (L-SSW)*. In last-state sensor windowing (L-SSW), a sensor is activated numerous times in a window specified by the Ai activity sensor. At times, according to A, the latest sensor state can be more descriptive than the frequency with which it occurs in a window [17]. The Vi feature vector can be calculated: for each Si sensor, when its last state is in an ON/OFF window, it shows 1/-1 in the Vi feature vector; otherwise, 0 (if not available) will be displayed. Some people have named this approach the latest window sensor for future reference. There may be a significant time interval between an event and previous events [25]. When there is a time delay, the use of sensor data in this section with the latest event may be small. This method has challenges for two or more people living in a smart home. A unit can include two-person sensor events.

*Feature Extraction.* Regardless of the sensor type, the HARS feature extraction step divides the sensor's information into fixed or variable-length time slices. Only one activity is labeled at each time slice. Since the activities are not always performed consecutively and are not uniform, a window may contain more than one activity [63]. Recognition of online training when a specific application performs ADL step by step is required to provide a person with home interventions or describe brief instructions on completing the task [27]. Each activity includes some continuous basic moves [64, 65], and usually, human activity can last several seconds, and several basic movements can be involved in one second. From the point of view of sensor signals, continuous motions are more related to smooth signals, and changes between base continuing motions can create significant signal value changes. These belongings of signals in activity recognition need feature extraction methods to capture the nature of tandem base motions and a combination of base motions [12]. In the previous section, we explained in detail about windows, and in the following, we will describe the methods of feature extraction, which can be either static or dynamic.  Table 5 shows the analysis of several important methods for feature extraction. Of course, it is worth noting that in addition to these basic methods, DL methods such as the convolutional method can be used directly to extract features or even a combination of two or more deep learning methods can be used.

*PCA.* One of the most popular features extraction techniques is PCA. This linear method converts main features (generally interdependent) into new features that are not interdependent and depend on the data scale. PCA is a statistical method that converts correlated variables into non-correlated ones [61]. In this method, the main components are not always easy to interpret. Filtering methods are fast and scalable and offer good computational complexity, ignoring class interaction. These new features are the main components. PCA's main idea is to restore the main features sorted according to their variance. The main components that reduce conflict are removed [22].

*LDA.* LDA has common goals with PCA, including finding a linear combination of variables that best represent the data and reducing computational costs [61]. Unlike PCA, this method minimizes internal class changes and separates classes [22]. However, this method also faces limitations, such as being dependent on a complex model and having low flexibility in dealing with complex datasets because it is linear. The LDA, on the other hand, needs a lot of data to classify, which creates a weakness in the performance of the HARS and does not work well in classification [62].

*Independent Component Analysis.* In the independent component analysis (ICA) method, randomly observed variables use the base function. The components in this method are statistically independent, and non-Gaussian data are used [66]. This method focuses on predicting essential features, and the probability distributions are statistically independent. Although this method was initially proposed to solve the blind source separation (BSS) problem, it has become popular today for feature extraction [22, 67]. ICA features are very useful in describing local features [68]. Also, ICA is computationally expensive and therefore not currently available for wearable online algorithms.

*Factor Analysis.* Factor analysis (FA) is another feature extraction method in HAR that groups the main features based on the correlation. FA represents each group of strongly correlated traits but has little correlation with other groups' characteristics by some factors [22]. In the FA method, examining the factors and finding the most effective ones is considered a challenge. Due to human behaviors' complexity, comprehensive influencing factors should be achieved through exploratory work [10].

*Extraction of Deep Learning-Based Features.* One of the most recently used feature extraction methods is extracting deep learning-based features (DLF) using machine learning methods [66]. With this technique, the salient features of raw data can be extracted automatically, without depending on handcrafted features [12]. In the HARS, complex activities are hierarchically unstable [69], which means people do the same activities in different procedures. In some cases, simple activity is the beginning of a complex activity. For example, running and jogging are performed dependently on the individual's oldness and fitness condition [70, 71], and the activity may not be recognizable. Classical machine learning (SVM, KNN, K-means, and so on) requires feature engineering to execute optimally. The important deep learning methods have recently been proposed to detect human activity, categorized into restricted Boltzmann machines, deep autoencoder, sparse coding, CNN [72], and RNNs [67, 73, 74]. DL's most crucial benefit is the ability to learn from unauthorized automatically and, in some cases, unlabeled raw data. However, these methods offer different capacities for processing sensor current. The challenge of one of the methods is that it takes a lot of computational time to reach an optimal solution because it sets many parameters [66].

*Feature Selection*. The feature extraction step aims to reduce the extracted features and select a subset of practical features. Classification algorithms need feature representative vector to differentiate among samples. In order to improve the performance of classification and recognition methods, inappropriate features should be avoided, which depends on the feature selection method. As a result, it leads to high dimensions and reduced class performance, so choosing a limited number of features with the desired ability is essential [41]. The feature selection procedure is specified as a searching manner under the appropriate set of features [75]. Feature selection is necessary for the HARS to reduce the complexity of calculations, time, and recognition accuracy [22]. Feature selection techniques minimize dimensions by removing some of the main features, while feature conversion methods map the main features to a low-dimensional subspace [76]. In general, feature selection methods are divided into three types: filter, wrapper, and hybrid, which are described below.

*Filter Methods.* Filter methods work directly on the dataset, using inherent feature details. In filter mode, it does not use classification, and second, features are ranked based on their values. Then, the selection operation is performed from a group of features.

*Wrapper Methods.* This method, unlike filtering methods, uses classification to select features. It often has better results in HAR than the filtering method.

*Hybrid Methods.* This method combines machine learning and DL methods based on internal parameters. The validation process step is not required in the feature selection process [27, 77].

#### 6.2.2. Categorization and Recognition

After preprocessing, feature selection and extraction operations are performed on the raw data, and the output must be entered into the activity classification and recognition algorithms [78]. In the HARS, input data patterns with activities (classes) are examined. Wearable sensor HARS can be classified into two stages. The first step is to choose a learning approach that can be supervised, semisupervised, or unsupervised. Secondly, it should be considered whether the system is online or offline. As the signals are received in the online mode, the activity must be recognized to provide the relevant service when it is necessary. The offline mode requires more time to recognize because the computations are high and unsuitable for real-time systems. There are three main approaches to machine learning techniques: supervised, unsupervised, and semisupervised. In supervised learning, there are various methods for learning such as SVM, least squares (LS), KNN, artificial neural network (ANN), DT, random forest (RF), and QDA [10]. The unsupervised approach includes Gaussian mixture model (GMM), K-means clustering, and HMM, automatically obtaining labels from the data [17, 22].

On the other hand, probabilistic and statistical classifications such as Naïve Bayes (NB**)** [79], SVM, conditional random field (CRF) [79], HMM, and dynamic Bayesian network (DBN) provide a valuable framework for accessing temporal and unreliable information. Problems such as performing certain activities differently by different people and uncertainty in the activity duration create issues [80].  Table 6 analyzes important methods for HAR.

*Supervised Learning.* The most critical problem with supervised classification is that the need for targeted training to create the most accurate model is essential considering the input and output available. On the other hand, to increase the accuracy of diagnosis in HARS, the lifestyle of the elderly should be followed, and the process should not be based only on a specific method or condition and device. The elderly are a particular group in HARS that most systems recognize only a few activities. Initial training should be done so that a new activity performed after training should be correctly identified, so in the laboratory environment, learning processes should be done carefully. Therefore, HARS should be in a controlled environment under an experienced team's supervision and a standard and public dataset [77]. As mentioned earlier, in activity recognition in smart homes, we see regular activity to monitor healthcare and find changes in people's patterns and lifestyles [81]. Because there are different methods to activity recognition in related fields, it is necessary to provide a review of each method according to the existing programs and challenges. Various categories have been proposed for the classification of these approaches, including the fact that it can be said that the classification methods are divided into three general categories, which are top-down, bottom-up, and combined.

In bottom-up activity recognition methods, a learning activity model uses the sensor's data through data mining and machine learning techniques and attempts to recognize activities. These methods are distributed into three categories: probability-based, similarity-based, and integration-based [33]. In the following, we will review the handcrafted methods for supervised learning.

**Figure 13:**
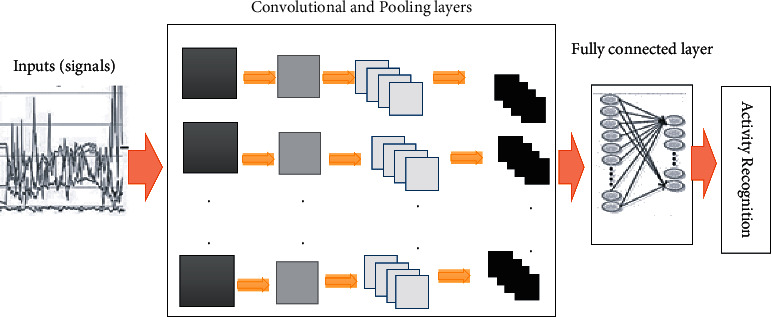
CNN layers for HAR [76].

(i) ***KNN***. KNN is one of the most popular activity recognition methods that does not involve a learning process and does not require information storage. This method is supervised because labeled datasets are used. The new sample class is recognized with similarity, which is identified by the voting operation among neighbors. A neighbor's distance of instance is calculated using distance measurements such as Euclidean distance. This method is the base for comparing the accuracy of classifiers. HAR in this method is at a high level of accuracy. The results of the classification will be satisfactory. The calculation time in assigning a new sample to the relevant class is relatively high. This method performs an inclusive experimental study of time series classification difficulties. K can take different values, which must be chosen carefully because too large or small of a value can lower the detection accuracy. So, when considering K to be 1, it looks like the SVM method [12]. Experiments show that KNN classification has a good performance compared to many supervised classification algorithms.
(ii) ***SVM***. The SVM is a classification method in which the raw time series sample is used directly as the SVM input. The cross-validation technique adjusts the SVM parameters [12, 82]. This method uses statistical learning theory, which tries to increase separators and often uses the radial basis function (RBF) to perform better. The SVM classification function can be expressed as follows:
  (1) $$\begin{array}{c} f\left(x\right)=\sum_{i=1}^{l}y_{l}a_{l}k\left(x_{1,}x\right)+b, \end{array}$$
where *K*(*x*_*l*_, *x*) is a kernel function used to measure (*x*_*l*_, *x*) training vectors. There are several kernel functions. One of the points in this method is that it transfers data to a larger space for better separation. One of the SVM characteristics is based on two classes, extended to multiclass solutions. So, SVM can be converted to non-linear classification to increase performance. When classifying learned activities and identifying new unknown activities with strong generalization capabilities [76], recognizing intricate movement patterns and visual patterns is achieved. Also, in this video method, local time space features local record events. They can be adapted to the size, frequency, and speed of moving patterns and recognize complex motion patterns [70]. This method's key challenge is that it does not perform well on large-scale data and is costly. In other words, the main challenges of classic SVM are high training time. These forms challenge the use of SVM in activity recognition systems, which generally have a large dataset [71].
(iii) ***DT***. In the DT method, a set of features must be selected correctly to have high accuracy for recognition. This method uses a sliding window [83], which has an excellent computational performance. Some research work using other sensors in conjunction with inertial sensors uses conceptual information to improve diagnostic accuracy [76].
(iv) ***RF***. RF includes a combination of decision trees that select essential features to perform the classification operation [82]. The RF method improves the DT method, i.e., it is a combination of several trees. Based on the trees' majority vote, a decision is made about the new sample, and the activity is recognized. This method requires more examples for training than DT [22].
(v) ***QDA***. In the QDA method, noise injection is proposed to progress HAR models. This method's impression is to use a small dataset to create general diagnostic models for e-health; expansion of the space enclosed by the training data is done by using noise, which injects noise to improve the actual positive recognition rate. The results show that this method increases the accuracy and reduces the false-positive rates. Besides, experiments have been performed using various training data sizes to show that the actual positive rate progress will be enormous if the main training set is short. In other words, noise injection is used to expand the area covered by training data. Thus, models are taught to use it when it is less vulnerable and more general as conditions change and recognition rates improve, specifically if the training data are low. Therefore, training models may be less susceptible to evolving circumstances and more accurate. This method can be used with any time series of data, but the data collection phase can still be unbearable [52]. This method is accurate, fast, and independent of the model and uses quadratic levels for class separation. Therefore, it uses any classification algorithm. However, personalization cannot improve the recognition rate when the user-independent model used in the first stage of the diagnostic process is inaccurate. In fact, for a large dataset, it may not be efficient enough for activity recognition [84].
(vi) ***Deep Learning-Based Classification***. Deep learning-based classification (DLC) is a learning type aimed at high-level abstract data models recently. In DLC, a deep multilayered architecture is created for automated feature design [48, 85]. The deep architecture layers make a non-linear change at the previous layer's output. The data are provided by a hierarchy of features from the bottom to the top through DLC models. Well-known DLC models include CNN, DBN, RNN, and autoencoders. Due to information labels, DL models can be performed in both methods: supervised and unsupervised. DL methods have made significant progress in various fields, including natural language processing. However, the HAR still needs to be researched to achieve the best possible performance [12, 86]. Choosing the correct DLC method for HAR is difficult.
Tasks that promote DLC almost always provide better system performance and rarely include discovering its seemingly desirable parameters. It is unclear how this efficiency contrasts with the mean during parameter exploration. While there is some in-depth exploration of models for various practical states in HAR, there is still a shortage or absence of organized investigation of DLC capabilities. The researchers report space parameter discovery in early tests but typically forget details. The inclusive procedure is unclear and complicated, and questions such as which parameter has the most significant impact on performance remain unanswered [49]. Deep NNs represent non-linear [23] conversion sequences to network input data. This rule can be followed, and a network with a hidden N layer is an N-layer network. Activation function and linear conversion are the primary units of each hidden layer. In this paper, we will discuss some methods of DLC.
(vii) ***RNN***. Most RNN-based models are used to manage space-time variable-length inputs. RNN is one of the NN methods suitable for HAR based on time series data. In HARS, there is a possibility that the data in each window are related to the previous window, which supports this method. Therefore, one of the places of interest of this method is that the final output of each layer is stored and helps a lot in the subsequent detection, i.e., the input of the current layer is a combination of the result of this layer in the previous time and the output of the previous layer [87]. RNN consists of non-linear units and performs learning based on consecutive inputs and time dynamics. Thus, they dominate the limitations of CNNs expected to have a fixed input length [88]. However, propagating the gradient down through many loop network layers can easily reason for gradient vanishing and exploding. Therefore, modeling long-term dynamics is difficult [87]. Another problem with using RNN is that it has a high time complexity due to the high volume of parameter updates, which requires methods that reduce computation time [76].
(viii) ***LSTM*.** LSTM types are more popular than RNN models because of the traceable learning structure. LSTMs are used for the variability of activities, and each human activity is temporarily displayed. Such temporary information complements spatial features and is serious about efficiency [89]. Each LSTM unit contains numerous memory cells that store data for a little time [11]. LSTM content is suitable for displaying complicated relationships when they may be in a wide range. Some gateway units control the memory cell contents containing the information flow inside and outside the cell. Managing them also helps avoid spurious gradient updates, typically in RNN training, when input time is extended. This feature allows us to stack many layers to learn the input's complex dynamics in different ranges. LSTM layer above the individual trajectory procedures is the first phase of its ordered model. This step is designed to model actions at the personal level, their temporal evolution. This stage is responsible for modeling activities based on time series [88]. LSTM was proposed to exploit the temporal dependencies of motion data. This architecture is reversible because some within-network connectivity makes a straight rotation, in which the present time step *t* considers the network modes at the last time step *t*_1_. LSTM cells are designed to counteract reduced gradients if many layers propagate fault derivatives via “time“ in open return networks. Each LSTM cell holds an inner mode check, that is, “memory.“ The learning operation is based on input and past status; the system can store information in several steps. According to (2), the update of the LSTM layer is done.
  (2) $$\begin{array}{c} i_{t}=\sigma_{i}\left(W_{ai}a_{t}+W_{hi}h_{t-1}W_{ci}c_{t-1}+b_{i}\right),\\ f_{t}=\sigma_{f}\left(W_{af}a_{t}+W_{hf}h_{t-1}+W_{cf}c_{t-1}+b_{f}\right),\\ c_{t}=f_{t}c_{t-1}+i_{t}\sigma_{c}\left(W_{ac}a_{t}+W_{hc}h_{t-1}+b_{c}\right),\\ o_{t}=\sigma_{o}\left(W_{ao}a_{t}+W_{ho}h_{t-1}+W_{co}c_{t}+b_{o}\right),\\ h_{t}=o_{t}\sigma_{h}\left(c_{t}\right), \end{array}$$
where *i*, *f*, *o*, and *c* are the input gate, the forget gate, the output gate, and the cell activation vectors; all of them are of the same size, all *h* are hidden values, and all *σ* are non-linear functions. Input, output, and forget gates have the role of control, so we have considered *i*_*t*_, *o*_*t*_, and *f*_*t*_ coefficients for them in time *t*, respectively. The term *a*_t_ is the input to the memory cell layer at time *t*. *W*_*ai*_, *W*_*hi*_, *W*_*ci*_, *W*_*af*_, *W*_*hf*_, *W*_*cf*_, *W*_*ac*_, *W*_*hc*_, *W*_*ao*_, *W*_*ho*_, *W*_*co*_ are the matrixes of weight and relationships, in which *W*_*ai*_ is the input-input gate matrix, *W*_*hi*_ is the hidden-input gate matrix, and the rest of W are named this way. *b*_*i*_, *b*_*f*_, *b*_*c*_,  and *b*_*o*_ are bias vectors. *c*_*t*−1_ is cell output at the previous time stage, and *c*_*t*_ is the state of memory at time *t*.
Although LSTM performs promisingly and solves the vanishing/exploding gradient challenge of RNN [87] and overcomes backpropagation [90], it faces a complex structural challenge model. Also, memory cells that store time modes include multiple gateways to control information flow in and out of memory cells. It should be noted that its complexity influences this approach's training and decoding operations. Another problem of LSTM is the difficulty in accelerating decoding [91].
(ix) ***DBN***. The DBN uses supervised and unsupervised methods to understand features in a hierarchical architecture to classify and identify patterns. The technique is trained in two stages. The first step is pretraining, which is used to train basic parameters in an unsupervised layerwise manner. In contrast, the fine-tuning step uses a supervised strategy to adjust the parameters according to the labeled samples and softmax. A DBN is a possible production model consisting of several layers of hidden units that typically use ANN. In this approach, there are concepts related to each other so that low-level concepts lead to higher-level concepts. So, this approach is a statistical model with specific groups of concepts [92]. Like the convolutional method, this method first produces a set of *r* *∗* *D* matrix samples. The DBN input is the mean of the *r* samples' signals in the *r* *∗* *D* matrix. A combination of two KNN classifiers (*K* = 1) and a perceptron neural network is used [12]. DBN solves quickly the problem of falling at the local minimum point. This method's challenge is the balance between learning rate and learning accuracy. DBN and autoencoder have recently been used in unsupervised learning, consisting of several hidden single layers. DBNs are useful in extracting features and finding massive data patterns. Deep production models are also more robust against overfitting problems than discriminative models. Therefore, researchers tend to extract unlabeled data features because it is easy and inexpensive [93].
(x) ***CNN***. Although the CNN approach is mainly used for image classification, it performs well in time series signals received from wearable sensors and generates high-value features [61]. The deep architecture of this approach can extract special features from these signals representing a high level of abstraction [94]. Using information marked through supervised learning, learned features have more optional power in an integrated model, and learning and feature recognition are mutually improved. All these exclusive benefits of CNN encourage it to be superior to other activity recognition methods. Each CNN holds at least one temporal convolutional layer, a pooling layer, and at minimum one fully connected layer before a classifier.  Figure 13 shows the general architecture of this neural network. A key feature of CNN is the management of alternately various processing units (e.g., convolution, pooling, sigmoid/tangent, sigmoid/hyperbolic squashing, rectifier, and normalize). Such different processing units can indicate the effectiveness of local signals. The deep architecture allows the multiple layers of this processing unit to be stacked so that this DLC model can determine the number of signals at various levels. Thus, the features extracted by the CNN are activity-dependent and non-manual, and features have more detection influence [12, 95]. CNN has great potential for detecting different prominent templates of signals. In particular, the processing units at the lower layers provide the signals' local prominence (to determine the nature of any fundamental move in HAR). The higher layers' processing units deliver prominent signals to the high level (to specify a combination feature of some base actions).

It should be noted that each layer may have many pooling or convolution operators (characterized by parameters), so several significant patterns from various aspects are commonly measured in the CNN. An unchanging interpretation is found after these operators apply the same parameters to resident signals (or mapping) in different time segments. As a result, only the noticeable pattern of signals should be considered, rather than their position or scale. However, in HAR, CNN faces challenges that include the following:

- Processing units in CNN need to be used for a length of temporal dimensions.
- Sharing or integrating CNN units between different sensors.
- Selecting a smaller window size leads to higher computational costs [12].

In general, the use of CNN for HAR is performed with the dynamic features. According to research, compared to some multilayer neural networks and traditional networks, CNN has performed better in HAR, especially in the dynamic state [8]. One of the problems of this method is that it requires a lot of memory and high computational complexity [76]. CNN has two advantages: it has a local dependency and does not change the scale. Local dependence means that close signals in the HAR are likely to correlate, while lack of scalability refers to scale invariance for different paces or frequencies. Due to CNN's effectiveness, most of the work reviewed focuses on this area [10]. Also, deep convolution features have good generalization [13].

- ***Unsupervised Learning***.
- ***GMM***. GMM is a probabilistic clustering method that is generally unsupervised and widely used and optimizes the proportion between the data and the parametric distribution [96]. The whole data are modeled with a combination of several distributions. GMMs work better while data are scattered and highly varied than methods such as K-means [62].
- Contrasting standard probabilistic models with data approximation and a single component density, GMM enjoys the limit's Gaussian component's total weight density. GMM contains parameters that must be checked and specified by the expectation-maximization (EM) algorithm, and various activities are detected. Based on the highest probability, classification operations are performed in this classifier. The initial value of the EM algorithm will have a significant effect on GMM, and on the other hand, there is no possibility of convergence in GMM. But research shows that GMMs have been used for HAR using the Gaussian distribution [97].
- ***K-Means***. K-means method can categorize *n* objects in a k-class. This clustering means finding spherical clusters of similar sizes while the sensor data are not spherical and noisy. Hence, the K-means method shows a precise distinction. Accuracy and other criteria come down when *k* is less than a value because the main clusters merge. For example, two clusters related to walking and running are merged [97]. The K-means method reduces the size of the total variance distortion within the cluster as a cost function. The cluster centers' detection is repeated, passing the data to the target cluster with their distance (e.g., Euclid) from the cluster's center until it converges. One of the problems of K-means is that it can have low cluster overlap and does not determine data density. Therefore, it cannot assess uncertainty about data categorization, especially in overlapping areas. The K-means algorithm has advantages such as low computational complexity, high performance for large datasets, and high linearity of time complexity. However, the clustering and iteration time results depend on the initial centers of clusters, and the algorithm can operate very slowly to converge with the wrong initialization [98].
- ***HMM***. One method in which the current state is dependent on the previous state and involves a limited number of states is HMM, which operates in discrete time. Considering that each Markov chain is compatible with consecutive data such as signals received from wearable sensors and is dynamic [14], this is an excellent method for HAR. This method tries to control sequences. The method's problem is that there is a possibility of non-convergence, and the initialization of the EM algorithm must be considered. HMM has a high performance for detecting short-term activities. It has the potential to hold scheduling information on out-of-range data. This method helps recognize time patterns, identify interleaved activities, and predict activity labels when moving slowly from one activity to another. However, the HMM learning model, unlike CRF, may not have long-term dependence on the observed sensors due to its strong independence. However, in the CRF learning model, there is an overfitting probability. In general, approaches that can model temporal relationships are graphical models. They can model complex activities. Researchers have designed the exploration of repetitive sensors to recognize sequential, cross, and simultaneous activities and scalability and noise resistance to recognizing single and multiple activities [33]. One of the critical drawbacks of HMM is that it sometimes recognizes a sequence that contains more than one activity as active. Hence, it is not suitable for complex activities.
- ***Semisupervised Learning***. One of the challenges of learning with the supervisor is that a large amount of labeled data is required, and on the other hand, data labeling is expensive [99]. As it turns out, collecting unlabeled data is accessible. An approach can be used that combines unlabeled and labeled data; semisupervised learning has the same idea. Since producing a large volume of activity samples that do not need to be labeled is an easy task, the semimonitored method is used in this area. It also uses labeled data and has a good performance in HAR [62, 100]. Semisupervised learning works better than fully supervised learning with unlabeled data [41]. In practice, the EM algorithm is used to identify the composite components [62, 101]. Naturally, in semisupervisory learning, it is supposed that a significant amount of unlabeled training data is also available with the small set of labeled training data [61] and minimizes monitoring while still maintaining a competitive detection performance. Many classical semisupervised learning methods include generative, self-training, co-training, multiview learning and Gaussian process models, S3VM, some graph-based methods [42], and deep learning methods that have been introduced over the past decades [62]. Here we describe four famous methods.
- ***Self-Training***. Self-training is a wrapper algorithm that repeatedly uses a supervised learning method. A supervised classifier is first taught with a small amount of labeled data. The classifier is then used to classify unlabeled data. Part of the unlabeled data is labeled according to the current decision performance in each iteration. Typically, the most reliable predictions are added to the labeled training set. The classifier is retrained, and the self-training process is repeated [62]. Although self-training works well in some cases, it is still challenging to analyze because it is a wrapper algorithm.
- ***Co-Training***. Co-training follows repeated self-training. Simultaneously, the goal is to progress self-training by strengthening the training procedure with one origin of the knowledge. Therefore, acceleration and infrared sets can first train two distinct classifiers. The classifiers prepare each other for reliable predictions by reinforcing each other's training sets. Classifiers are then retrained with a collection of labeled data, and this operation is continuously repetitive. In this method, two points should be considered. First, the features should be separated into two categories. Second, these two categories of features should not be related to each other, and both types should be reliable. The data points with the high confidence of one classifier are separated, and the samples are distributed equally to the other classifiers [36].
- Co-training has performed well in learning operations with unlabeled data, but it may not be well-received due to limitations. The first limitation is sufficient data under two separate and independent subsets, which is sometimes problematic. On the other hand, each classifier's labeling reliability should be carefully measured to determine which sample should be labeled. Sometimes, this measurement process is very time-consuming, ensuring each classifier's labeling is necessary to combine both classifiers' output [61].
- ***Generative***. Generative models are considered to be the oldest method of semisupervisory learning. This model assumes a recognizable composite distribution *p* (*x*|*y*) such that the model *p* (*x*|*y*) is represented as *p* (*x*) *p* (*x*|*y*). Distributions can be hybrid Gaussian or similar distributions—generative model core for semisupervised learning using large amounts of unlabeled data to identify composite components. An unlabeled data point for each class is sufficient to determine the compositional distribution and fully recognizes missing data. Generative models are not always suitable for all semisupervised learning tasks. There is not always an identifiable hybrid distribution that can help build the generative model. For example, a multivariate Gaussian or Bernoulli hybrid is not recognizable. Therefore, the data generated by this type of model will not be suitable for using the generative model method [45]. Most production approaches have been inflexible, inefficient, or scalable.
- ***Deep Learning-Based Semisupervised Model***. Deep learning-based semisupervised model (DLS) has been introduced more recently with the development of DL methods. In general, these types of strategies have learned ideas from the generative model. Since most well-known DLC methods such as CNN and LSTM can also be considered generative and discriminator models, it is not surprising to know that they can learn directly from unlabeled data. This part is usually implemented with a proper structure design and a complex loss function that can control unlabeled and labeled data points. Pretrained models are then retrained with labeled data [62]. As a result, in this approach, the cost of learning is high, and we also face many parameters.

## 7. Evaluation and Testing

This section first introduces the criteria for evaluating the automatic HAR in smart systems. We then qualitatively assess the macro-activity recognition approaches categorized based on their key characteristics. Finally, we will analyze these results.

### 7.1. Criterion for Evaluation

In the following, we describe the criteria for HAR approaches' evaluation [77].

(i) ***Accuracy***. It indicates trust in the system evaluating the HAR. Accuracy is the number of classified activities correctly (diagnosed) to the total number of activities classified:
  (3) $$\begin{array}{c} \text{accuracy}=\frac{\text{items classified correctly}}{\text{all items classified}}. \end{array}$$
(ii) ***Precision and Recall***. The total number of correctly identified samples known as recall and accuracy in the HARS are expressed in (4) and (5), respectively.
  (4) $$\begin{array}{c} \text{Recall}=\frac{\text{TP}}{\text{TP}+\text{FN}}, \end{array}$$
  (5) $$\begin{array}{c} \text{precision}=\frac{\text{TP}}{\text{TP}+\text{FP}}, \end{array}$$
where TP And FP are the number of true positives and false positives, respectively, and FN is the number of false negatives [36].
(iii) ***F-Measure*.** The classification accuracy is often high for weak classifiers, so it is not a good criterion. Therefore, another standard criterion called *F*-measure is used in addition to this criterion. The *F*_1_ result includes two precision and recall measures that express the system's trust under evaluation to identify the agent's activities. The accuracy of known activity samples is calculated using the *F*-measure, shown in (6), and *F*_1_ in (7) is used for this criterion by weight injection:
  (6) $$\begin{array}{c} F-\text{measure}=\frac{2*\text{precision}*\text{recall}}{\text{precision}+\text{recall}}, \end{array}$$
  (7) $$\begin{array}{c} F_{1}-\sum_{i}2\times w_{i}\frac{{\text{precision}}_{\text{i}}\times {\text{recall}}_{\text{i}}}{{\text{precision}}_{i}+{\text{recall}}_{i}}, \end{array}$$
where *i* is the class index and *w*_*i*_ is the ratio of class *i* in all samples (*w*_*i*_=*n*_*i*_/*N* where *n*_i_ is the number of samples in *i*th class and N is the total number of all samples). Precision_i_ is the ratio of class *i* correctly predicted on all predicted samples. Recall_i_ is the sample ratio of class *i* that is correctly predicted on all correct samples [36, 97].
(iv) ***User Acceptance***. Consider that that user acceptance is necessary because the HARS, on the one hand, is an unusual thing in the user's daily life. In particular, devices that receive information from the user must be connected to the user (wearable sensors). Therefore, this criterion should be evaluated with a questionnaire, and users' opinions such as invasion, installation in the living environment, and the maintenance process's complexity [102] should be measured.
(v) ***Time Complexity in Recognition (TCR)***. It measures the time elapsed from the moment a user initiates an activity until the system recognizes it.
(vi) ***Time Complexity in Modeling (TCM)***. It is defined as the time it takes for modeling on the training dataset.
(vii) ***Installation Complexity***. This criterion deals with the difficulty and complexity of setting up a HARS in the smart home and the intended user or users. A function can be considered to calculate this complexity.
(viii) ***Interoperability***. This criterion deals with the integration and interaction of systems and measures their difficulty. The evaluation uses a questionnaire based on this criterion, including access to APIs and documents, license design, testing tools, and portability.

In addition to the mentioned criteria, reliability, adaptability, generality, complexity, simplicity, and scalability are also used for evaluation.

### 7.2. Evaluation and Discussion

Table 7 compares the important different methods of HAR based on the evaluation criteria introduced in this section. We have divided HAR methods into parametric and non-parametric, involving supervised and unsupervised learning. Informed-SVM, DT, QDA, KNN, and RF are supervised and nonparametric methods. k-means is an unsupervised and nonparametric method. GMM, HMM, and DBN are unsupervised and parametric methods. Uninformed-SVM, RNN, LSTM, and CNN are supervised and parametric methods. We have used “–” “low,” “medium,” “high,” and “very high” to evaluate each method according to accuracy, TCM, TCR, and generalization criteria.

Statistical learning methods for HAR, such as running, walking, and so on, are essential for using the KNN classifier. These methods use handcrafted features, which is a crucial challenge because they cannot accurately distinguish different activities (simple and complex). Feature extraction methods such as symbolic representation, raw data statistics, and conversion coding are widely used in the HAR [103]. Still, they are exploratory methods and need expert knowledge to design features. As you can see, the deep structure has improved learning, notwithstanding the time essential to reach maximum accuracy. It is appropriate for complex, large-scale HAR problems where sensor fusion is needed, but these methods need strictly labeled data. Interpreting time series data from sensors such as accelerometers or gyroscopes is much more complex than data from other sensors such as cameras. An expert takes a lot of effort to accurately split and label an activity using a long sequence of time series data. Deep learning methods, which are part of the parametric category, have high accuracy and, on the other hand, high training time complexity. LSTM has higher accuracy than the rest due to traceable learning, which also uses the previous steps. This method is suitable for complex activities and has high generalization power, but the parameters to be learned are very high due to high computational complexity [8]. RNN time complexity is less than LSTM because it has fewer parameters, but LSTM is more accurate.

On the other hand, RNN has less generalization power than LSTM due to its two main problems (vanishing/exploding gradient) [76]. CNN has high generalizability and accuracy and will have higher computations in the smaller window and higher time complexity [68, 104]; also, CNN has notably compressed the performance of HAR due to their rich representation influence. Compared with other networks, such as CNN, restricted Boltzmann machine (RBM) [104], and autoencoder (AE), the structure of the LSTM makes it especially good at solving problems containing time series. SVM, a supervised learning method, can be parametric, and its parameters can be computed, or its parameters can be manually specified at the beginning. Parametric SVM has high accuracy, but the training time complexity will be higher [82]. In non-parametric and supervised methods, KNN is a simple method and has better performance and increased accuracy than some learning methods. It does not take time for modeling and has high generalizability, but the complexity of time during the diagnosis is high [22]. The results showed that the KNN method outdone other methods in most recognition of activities. Support vector machine (SVM) is another special algorithm. However, the QDA method has high accuracy (due to noise injection) and low time complexity for training and diagnosis, but low generalizability is a crucial challenge [105]. RF has higher accuracy and generalizability than DT, but its temporal complexity is more elevated than DT and requires a large amount of labeled data to achieve approved efficiency [22]. K-means is a non-parametric and unsupervised method, although a simple process, without training and with high generalizability [106]. But K-means has low accuracy. The complexity of recognition time is very high, especially when the number of dimensions is high and suitable for numerical data and does not apply to qualitative data [8, 107]. This method is appropriate while datasets lacking labels are employed, or the measure of similarity/dissimilarity between classes is the primary outcome. Although DBN has high generalization accuracy and power, it also has a relatively high time complexity because it belongs to the parametric category. The structure of a DBN requires fitting sets for the hyperparameters so that performance of a DBN may differ and can be degraded depending on the configurations.

GMM has high time complexity and low accuracy and does not have high generalizability. HMM is a balanced method in terms of the mentioned criteria.

## 8. Conclusion and Future Research Directions

HAR is an important research subject in healthcare in the present age. The need to analyze time series data to HAR has increased the attention of researchers in this field. In HAR, it is crucial to select efficient features from time series data. There are many challenges in this regard, and on the other hand, using a method that categorizes actions with high accuracy is a need in this area. Therefore, in this paper, we examine all aspects of the SHCS, including the activity recognition component, and provide an architecture for the SHCS. The applications of activity recognition and its key challenges were reviewed. Then, the types of macro-methods in feature extraction, feature selection, and classification were compared. Each of the methods was examined separately. Finally, after the methods' general categorization, a qualitative comparison was performed based on some essential criteria. We also reviewed and analyzed popular datasets and categorized and explored different types of sensors. Considering that this paper has examined all aspects of HAR, it will be helpful for researchers in the area. Although this paper examines the various elements of HAR, it is possible to categorize HAR methods from other perspectives and provide a comprehensive architecture for HAR, which we will address in future work. Other qualitative criteria can also be supplied for HAR, and then methods can be reviewed. According to the studied methods, deep learning methods have a special place that will need further study under one paper.

According to the most prominent research work surveyed, the number of sensors, their type, and location are significant; future papers will address these issues. Then, a comparison is made between the combinations of sensors and locations to categorize the dataset and take steps to create a more appropriate dataset. Then, it is possible to implement the sensor network in the best natural environment and ultimately achieve high activity recognition accuracy. Soon the world's population is aging, and their healthcare is essential. It is necessary to consider the data set based on the activities of the elderly that activities are analyzed to increase the accuracy of recognition and to be able to control the elderly remotely. This resolve leads to an increase in the standard of living of these people by considering solutions. We also identified and categorized most of the challenges, as seen in Section 5. In this area of HARS research, the main challenges include the complexity of some activities, online HARS, and flexibility of activities. Therefore, in future works, we will review the activities separately. Analyzing the activities makes it possible to identify which complex activities are a combination of simple activities, which will help in the proposed modeling of activity recognition in the system. On the other hand, we will examine the different modes of an activity (which is performed by several different people or whether a person commits a specific activity in several different ways, i.e., flexible activity), and we will analyze the signals. In future work, to increase the performance of the online HARS, we will also focus more on fuzzy systems, return networks, and ontology. In order to improve the accuracy of the recognition, the combination of wearable cameras and sensors and the application of deep learning can also be examined separately, which is a good topic. We also have decided to present a book by expanding this paper.

## Figures and Tables

**Table 1:** Abbreviations and symbols.

Abbreviations and symbols	Description	Abbreviations and symbols	Description
3D	Three dimensions	ANN	Artificial neural network
ABW	Activity-based windowing	BSS	Blind source separation
ADL	Activities of daily living	CRF	Conditional random field
AFE	Analogue front end	DLC	Deep learning-based classification
CCA	Canonical correlation analysis	DLS	Deep learning-based semisupervised model
CFS		DLF	Depp learning-based features
CNN	Convolutional neural network	DBN	Dynamic Bayesian network
CPD	Point change detection	EM	Expectation-maximization
CSS	Contact switch sensors	FA	Factor analysis
DBN	Deep belief network	FP	False positives
DFT	Discrete Fourier transform	FN	The number of false negatives
DL	Deep learning	GMM	Gaussian mixture model
DT	Decision tree	ICA	Independent component analysis
HAR	Human activity recognition	LS	Least squares
HARS	Human activity recognition system	NB	Naïve Bayes
HMM	Hidden Markov model	RF	Random forest
IMU	Gyroscope, accelerometers, and magnetic sensors	RBF	Time complexity in modeling
KNN	K-nearest neighbor	RBM	Restricted Boltzmann machine
LDA	Linear discriminant analysis	SBHAR	Smartphone-based HAR
L-SSW	Last-state sensor windowing	TCM	Time complexity in modeling
LSTM	Long short-term memory	Radial basis function	TCR time complexity in recognition
MEMS	Microelectromechanical systems	*w*_*i*_	The ratio of class *i* in all samples
Mhealth	Mobile health	F	Freight gate
NN	Neural network	*i*_*t*_, *o*_*t*_ and *f*_*t*_	Input, output, and forget gates considered in time *t*, respectively
PCA	Principal component analysis	h (all)	Hidden values
PI	Passive infrared	Recall_i_	Sample ratio of class *i* that is correctly predicted on all correct samples
PN	Number of participants	K	Kernel function
PWM	Pulse width modulation	N	The total number of all samples
QDA	Quadratic discriminant analysis	Precision_i_	The ratio of an instance of class *i* that is correctly predicted on all predicted samples
REALDISP	REAListic sensor DISPlacement	*b*_*i*_, *b*_*f*_, *b*_*c*_ and *b*_*o*_	Bias vectors
RFID	Radio frequency identification	*c*_*t*−1_	Cell output at the previous time stage
RNN	Recurrent neural network	*W*_*ai*_, *W*_*hi*_, *W*_*ci*_, *W*_*af*_, *W*_*hf*_, *W*_*cf*_, *W*_*hi*_ is hidden-input gate matrix *W*_*ac*_, *W*_*hc*_, *W*_*ao*_, *W*_*ho*_, *W*_*co*_	Matrixes of weight: *W*_*ai*_ is input-input gate matrix, *W*_*hi*_ is hidden-input gate matrix, and the rest of the W is named in this way
STEW	Sensor dependency extension windowing	*c*_*t*_	The state of memory at time t
SDW	Sensor-dependent windowing	O	Output gate
SEW	Sensor event-based windowing	I	Input gate
SHCS	Smart healthcare system	C	Cell activation vectors
SVM	Support vector machine	*n*_i_	The number of samples in *i*th class
TBW	Time-based windowing	*a*_t_	Input to the memory cell layer at time *t*
TP	The number of true positives	All _*σ*_	Non-linear functions
TSW	Time slice-based windowing		

**Table 2:** Comparison of significant recent research work (surveys).

ID	References	Architecture for HAR	Challenges of classification of HAR	Approaches of HAR	Quality evaluation of approaches	Dataset analysis	Sensor system	Sensor types	Application classification	Number of tables	Number of figures	HARS component classification	Analysis of every component with table	Discussion
1	[10] (2017)	No	No	No	No	No	Yes	Yes	No	2	9	No	No	No
2	[3] (2019)	Yes	No	Yes	No	No	No	Yes	Yes	10	3	Yes	Some components	No
3	[12, 13] (2019)	Yes	Yes	Yes	No	Ye	No	Yes	Yes	5	2	No	No	Yes
4	[19] (2016)	No	No	Yes	No	No	No	Yes	No	6	1	No	Yes	Yes
5	[20] (2020)	Yes	Yes	Yes	No	Yes	No	No	Yes	11	6	Yes	Yes	No
6	[21] (2021)	No	Yes	Yes	No	Yes	No	No	No	4	2	No	Some components	Yes
7	[22] (2020)	No	No	Yes	No	Yes	Yes	Yes	Yes	13	6	No	Some components	Yes
8	**This paper**	Yes	Yes	Yes	Yes	Yes	Yes	Yes	Yes	7	13	Yes	Yes	Yes

**Table 3:** Details of the most popular sensor-based activity recognition datasets.

Dataset	PN	Channel number	Sensors			Frequency	Activities
			Type		Number		
**Opportunity**	4	113	Commercial RS485-networked XSense inertial measurement units (IMUs)		5	30 Hz	**Gestures:** open door 1, open door 2, close door 1, close door 2, open fridge, close fridge, open, dishwasher, close dishwasher, open drawer 1, close drawer 1, open drawer 2, close drawer 2, open drawer 3, close drawer 3, clean table, drink from cup, toggle switch, null **Complex:** relaxing, coffee time, early morning, cleanup, sandwich time **Simple:** stand, walk, sit, lie
			Commercial InertiaCube3 inertial sensors		2		
			Bluetooth acceleration sensors		12		
			3D-accelerometer		1		
			3D-gyroscope		1		
			3D magnetic		1		
**UCI**	—	—	Galaxy smartphone: three-axial linear acceleration and three-axial angular velocity		2	50 Hz	12 daily activities, namely, three static activities (standing, sitting, and lying), three dynamic activities (walking, going upstairs, and going downstairs), and the switch of 3 static activities (standing-sitting, sitting-standing, standing-lying, lying-sitting, standing-lying, lying-standing)
**DLAs**	23	—	Each sensor includes three-axis accelerometer and three-axis gyroscope		3	—	Walking, sitting, standing, and so on
**PAMAP2**	9	—	Colibri wireless inertial measurement units (IMUs)		3	100 Hz	16 activities Static activities such as standing, sitting, lying down, and ironing Dynamic activities such as walking, running, cycling, Nordic-walking, walking-upstairs, walking-downstairs, vacuum-cleaning, rope-jumping, cycling, and playing soccer
			Accelerometer		1		
			Heart rate monitor		1		
			Gyroscope		1		
			Magnetic		1		

**SBHAR**	30	—	Smartphone	Gyroscope	1	50 Hz	Three static activities such as standing, sitting, and lying down and three dynamic activities as walking, walking-upstairs, and walking-downstairs
				Accelerometer	2		

**MHealth**	10	—	Accelerometer		1	50 Hz	Standing still, sitting and relaxing, lying down, walking, climbing stairs, bending waist forward, front arm elevation, knee bending, cycling, jogging, running, and jumping front and back
			Gyroscope		1		
			Magnetic		1		
**WISDM**	29	—	Mobile phone: accelerometer		1	20 Hz	Sitting, jogging, standing, upstairs, downstairs, and walking

**REALDISP**	17	—	9 IMUs	3D-accelerometer	1	40 Hz	33 fitness activities
				3D-gyroscope	1		
				3D-magnetometer	1		
				4D-quaternion	1		
**MobiAct**	57	—	Smartphone	3D-accelerometer	1	20 Hz	Nine different types of ADLs: standing, walking, jogging, jumping, stairs up, stairs down, sit chair, car step in, car step out, and four different types of falls: forward-lying, front-knees-lying, sideward-lying, and back-sitting-chair
				3D-gyroscope	1		
				3D-orientation sensors	1		

**Table 4:** Analysis of various windowing methods for HAR.

Methods	Idea	Advantage	Disadvantage
**ABW**	The data stream of events is divided into windows at activity change detection points	(i) Suitable for labeling data. (ii) If valid points are detected, the accuracy of detection increases.	(i) Failure in activity recognition correctly (ii) Suitable for online recognition (iii) Complexity of calculations in finding practical separation points (iv) Inaccuracy in the boundaries of activities

**TBW**	Event data streams are divided into windows with fixed time intervals	(i) The simplicity of implementation.	(i) Choosing the right window length (ii) Extremely influential window length in decision making

**SDW**	The data are split into windows with the same number of sensor events, and the results of window time lengths vary from window to window	(i) This approach offers computational benefits over ABW. (ii) No need for sensor events to classify past sensor events.	(i) There may be a significant time interval between an event and the previous event (ii) Performance is low in the face of two or more residents in a smart home (iii) Giving equal importance to all data (iv) The possibility of having a window containing sensor events for a long time (v) The possibility of having a window containing sensor events related to the transfer between two activities

**SDEW**	The mutual information of the two sensors described earlier depends on the order in which a pair of sensors occurs in the entire data stream	(i) Uses multiple sensors to increase detection accuracy.	(i) The possibility of losing some dependence between the sensors (ii) In parallel activities and sensor events, one activity can be described for other information (iii) Dependence between sensors

**L-SSW**	In a window specified by the Ai event sensor, a sensor can be activated several times	(i) Sometimes, the latest sensor status, according to ei, can be more descriptive than the frequency at which it occurs in a window. (ii) Simplicity of calculations.	(i) There may be a significant time gap between an event and previous events (ii) Challenges more than one person living in a smart home

**Table 5:** Analysis of some well-known feature extraction methods of HARS.

Method	Idea	Advantage	Disadvantage
**PCA**	It is a linear method and consists of converting the main features (generally interdependent) into new features that are not interdependent and depend on the data's scale.	(i) Returns the main features to a low-dimensional space. (ii) Elimination of the central parts leads to lower variance and increased accuracy.	(i) The principal components are not always easy to interpret. (ii) Changes within the class.

**LDA**	Features extracted through linear conversion to find the variables' linear composition, which is the best representation of the data.	(i) Minimizes changes within the class relative to principal component analysis. (ii) Converts the main features to a new space with lower dimensions. (iii) Maximizes segregation between classes.	(i) Relies on a complex model containing the correct number of components. (ii) Limits flexibility when using complex datasets. (iii) Lack of covariance matrix within the same class. (iv) The possibility of insufficient data to estimate the conversions in the separation of classes.

**ICA**	This method finds independent components such as main features expressed as a linear combination of components.	(i) Solution to solve the problem of blind source separation. (ii) Effective for describing local features.	(i) Suitable for non-Gaussian data. (ii) Computationally expensive. (iii) Unsuitable for online algorithms.

**FA**	The main features can be grouped according to their correlation.	(i) The features of each group are strongly correlated. (ii) Quantitative communication between the features of different groups.	(i) Investigates the factors and finds the most effective ones.

**DLF**	The salient features of the raw sensor data can be extracted automatically, without relying on handcrafted features.	(i) Ability to automatically learn from unauthorized and, in some cases, unlabeled raw sensor data. (ii) These methods offer different capabilities for processing sensor current.	(i) Searches for optimal solutions. (ii) High calculation time due to setting the above parameters.

**Table 6:** Analysis of proposed methods for classification and activity recognition based on wearable sensors.

Approach	Disadvantage	Advantage	Idea	Method
**Supervised learning**	(i) High calculation time in assigning a new instance to the class. (ii) Selects the appropriate similarity recognition method.	(i) Relatively high classification accuracy. (ii) Conducts a comprehensive empirical review of time series classification issues. (iii) Simplicity. (iv) Good performance against a large number of supervised methods.	The principle of similarity between the training set and new examples is used to classify. The latest instance is assigned to the respective class by a majority vote of its closest neighbors.	**KNN**
	(i) Has two classes. (ii) The costly operation of building a training package on large-scale data. (iii) Possibility of low performance in large datasets. (iv) High training time. (vi) Ignores remote data. (vii) Low performance in the dataset with high noise.	(i) Linear separation in the specified space. (ii) Saves time on detection. (iii) Also suitable for low training data. (iv) Strong generalizability. (v) Suitable for complex activities. (vi) Sturdy against heteroscedastic noise.	This method uses statistical learning theory that maximizes the margin between the separator and the data.	**SVM**
	(i) The direct impact of the selected feature set on accuracy. (ii) The possibility of overfitting in the small dataset and the great depth of the tree. (iii) Requires high-volume datasets.	(i) They have an excellent computational performance. (ii) Data noise resistant. (iii) Efficiency for high-volume datasets. (iv) Suitability in cases where the dataset lacks values for all features.	The DT uses static features of time series data and focuses on the sliding window.	**DT**
	(i) Needs a lot of labeled data to achieve good performance. (ii) Low performance on low data.	(i) Improves the performance of the DT. (ii) Compatibility with multiclass problem. (iii) Important feature selection for classification.	Random forests contain a combination of decision trees and are based on the majority vote of each tree's different decisions.	**RF**
	(i) Challenges in the data collection phase. (ii) Inaccuracy in the user. (iii) Independent model leads to decrease in accuracy. (iv) Ability to reduce the accuracy of big data.	(i) Noise injection is provided to improve activity detection models. (ii) High accuracy and reduction of false-positive rates. (iii) Less vulnerability to changing conditions. (iv) Good generalizability. (v) Less vulnerability to changes in circumstances.	This method's idea is that to create general recognition models for e-health, a small main dataset is used, and the area covered by the dataset is expanded using noise.	**QDA**
	(i) Lack of details about the seemingly desirable parameters. (ii) A lack of systematic exploration of deep learning capabilities. (iii) Selects the appropriate method of deep learning.	(i) Provides high-level abstraction models in the data. (ii) High accuracy.	Deep learning has emerged as a learning model branch, creating a deep multilayered architecture for automated feature design.	**DLC**
	(i) Saves only one step before. (ii) High calculation. (ii) Vanishing. (iv) Exploding gradient. (v) Difficulty of long-term modeling dynamics.	(i) Compatible with variable-length input. (ii) Saves the previous step for higher accuracy.	Includes non-linear units with internal modes that can learn dynamic temporal behavior from a continuous input with arbitrary length.	**RNN**
	(i) High complexity of the model. (ii) Poor decoding efficiency. (iii) Training and decoding costs.	(i) Traceable learning. (ii) Suitable for a variety of activities. (iii) Suitable for modeling complicated time relationships. (iv) Suitable for group activities. (v) Suitable for counteracting the effects of reduced gradients.	At each step, the memory's content from the first layer contains differentiating information that describes the person's movement and past changes in his activity. Over time, cells learn to output, overwrite, or ignore their internal memory based on the current input and past state history, resulting in a system capable of storing information in hundreds of steps.	**LSTM**
	(i) A difficult balance between learning rate and learning accuracy.	(i) Better performance than the perceptron. (ii) Solves the problem of falling at the local minimum point. (iii) Finds massive data patterns. (iv) An effective solution to solve the problem of gradient fading. (v) Useful for the low training set.	As with the convection method, a set of matrix surface samples is first generated. Then, the average of the samples' signals in each matrix is used as the DBN input.	**DBN**
	(i) Processing units in the CNN need to be used. Length of temporal dimensions. (ii) Sharing or integrating CNN units between different sensors. (iii) Selecting a smaller step size in the window to increase the sample size leads to higher computational costs. (iv) Requires computational time and high memory.	(i) Learned features have more power. (ii) Effectiveness of local signals and local dependence. (iii) No change in scale.	It is based on a deep architecture and contains at least one temporal convolutional layer, one pooling layer, and one fully connected layer before a classifier.	**CNN**
**Unsupervised learning**	(i) Convergence is not guaranteed in many cases. Dependence on the initial evaluation of EM algorithm.	(i) Suitable for detecting most activities. (ii) Good performance in the face of sparse data with a high diversity.	A probabilistic method is generally used in unsupervised classification that uses the Gaussian component total weight density.	**GMM**
	(i) Poor performance in cluster overlap Uncertainty about data classification, especially in overlapping areas. Merges two different clusters when *k* is less than the actual value. (ii) Dependence of clustering results and iteration time on the initial centers of the clusters. The algorithm can be very slow to converge with wrong initialization.	(i) Reduces the size of the total variance distortion within the cluster as a cost function. (ii) Low computational complexity. (iii) High performance for large datasets. (iv) High linearity of temporal complexity.	An unsupervised classification method is known for clustering *n* samples into *k* classes. This method involves repeating the cluster centers' detection and then passing the data to the desired cluster according to their distance (for example, Euclid) from the cluster's center until it converges.	**K-means**
	(i) Poor performance in cluster overlap. (i) Uncertainty about data classification. (iii) Merges two different clusters when *k* is less than the actual value. (iv) Dependence of clustering results and iteration time on the initial centers of the clusters. (v) The algorithm can operate very slowly to converge with wrong initialization. (vi) Convergence is not guaranteed. (vii) Recognizes a sequence that includes more than one activity as an activity. (viii) Not suitable for complex activities	(i) A dynamic method. (ii) High performance for detecting short-term activities. (iii) Compatible with the sequential data model.	A Markov chain expresses a discrete-time random process involving a limited number of states whose current state depends on the former. In the case of HAR, each activity is represented by a mode.	**Markov**

**Semisupervised learning**	(i) It is difficult to analyze because it is a wrapper algorithm.	(i) Limited cost for labeling. (ii) Good performance in some cases.	It is a wrapper algorithm that frequently uses a supervised learning method. A supervised classifier is training for the first time, with a small amount of labeled data.	**Self-training**
	(i) The need for data samples that should be described by two subsets which are sufficient and redundant. (ii) Used in quantitative applications such as text classification. (iii) To determine which sample is to be labeled, each classifier's labeling reliability must be carefully measured. (iv) Sometimes, this measurement process is very time-consuming.	(i) An excellent approach to using unlabeled data to improve learning efficiency.	This method follows the process of repeated self-training. Simultaneously, the goal is to improve by strengthening the training process with one more source of information.	**Co-training**
	(i) There is not always an identifiable composite distribution that can help build the generative model. (ii) Not suitable for all semisupervised learning tasks.	(i) Detects missing data for the classification problem. (ii) No cost of data labeling by an expert.	The core of the generative model for semisupervised learning is large amounts of unlabeled data to identify composite components. Then, unlabeled data for each class are sufficient to determine the compositional distribution fully.	**Generative**
	(i) High learning cost. (ii) Many parameters.	(i) Can control unlabeled and labeled data points. (ii) Relatively high accuracy	Most well-known deep learning methods, such as CNN and LSTM, conceived the generative and discriminator models. It is not surprising to know that they can learn directly from unlabeled data.	**DLS**

**Table 7:** Qualitative comparison of macro-HAR methods with some of the mentioned criteria.

Methods			Accuracy	TCM	TCR	Generalization
**Non-parametric**	**Supervised**	Informed-SVM	Medium	Low	Low	High
		DT	Low	Medium	Medium	Low
		QDA	High	Low	Low	Low
		KNN	High	–	High	High
		RF	Medium	High	High	Medium
	**Unsupervised**	K-means	Low	–	Very High	High

**Parametric**	**Unsupervised**	DBN	High	High	Medium	High
		HMM	Medium	Medium	Medium	Medium
		GMM	Low	High	Medium	Medium
	**Supervised**	Uninformed-SVM	High	High	Low	High
		RNN	High	High	Medium	Medium
		LSTM	Very High	Very High	High	High
		CNN	High	High	Medium	High

## Data Availability

No data were used to support this study.
